# Molecular Simulation of Covalent Adaptable Networks and Vitrimers: A Review

**DOI:** 10.3390/polym16101373

**Published:** 2024-05-11

**Authors:** Argyrios V. Karatrantos, Olivier Couture, Channya Hesse, Daniel F. Schmidt

**Affiliations:** 1 Materials Research and Technology, Luxembourg Institute of Science and Technology, 5, Avenue des Hauts-Fourneaux, L-4362 Esch-sur-Alzette, Luxembourg; olivier.couture@list.lu (O.C.); channya.hesse@list.lu (C.H.); daniel.schmidt@list.lu (D.F.S.); 2 University of Luxembourg, 2, Avenue de l’Université, L-4365 Esch-sur-Alzette, Luxembourg

**Keywords:** covalent adaptable networks, vitrimers, dynamic bond exchange, topology, diffusion, mechanical properties, viscoelastic behavior, self-healing, nanoscale, force fields, atomistic modeling, coarse-grained models

## Abstract

Covalent adaptable networks and vitrimers are novel polymers with dynamic reversible bond exchange reactions for crosslinks, enabling them to modulate their properties between those of thermoplastics and thermosets. They have been gathering interest as materials for their recycling and self-healing properties. In this review, we discuss different molecular simulation efforts that have been used over the last decade to investigate and understand the nanoscale and molecular behaviors of covalent adaptable networks and vitrimers. In particular, molecular dynamics, Monte Carlo, and a hybrid of molecular dynamics and Monte Carlo approaches have been used to model the dynamic bond exchange reaction, which is the main mechanism of interest since it controls both the mechanical and rheological behaviors. The molecular simulation techniques presented yield sufficient results to investigate the structure and dynamics as well as the mechanical and rheological responses of such dynamic networks. The benefits of each method have been highlighted. The use of other tools such as theoretical models and machine learning has been included. We noticed, amongst the most prominent results, that stress relaxes as the bond exchange reaction happens, and that at temperatures higher than the glass transition temperature, the self-healing properties are better since more bond BERs are observed. The lifetime of dynamic covalent crosslinks follows, at moderate to high temperatures, an Arrhenius-like temperature dependence. We note the modeling of certain properties like the melt viscosity with glass transition temperature and the topology freezing transition temperature according to a behavior ruled by either the Williams–Landel–Ferry equation or the Arrhenius equation. Discrepancies between the behavior in dissociative and associative covalent adaptable networks are discussed. We conclude by stating which material parameters and atomistic factors, at the nanoscale, have not yet been taken into account and are lacking in the current literature.

## 1. Introduction

**Figure 1:**
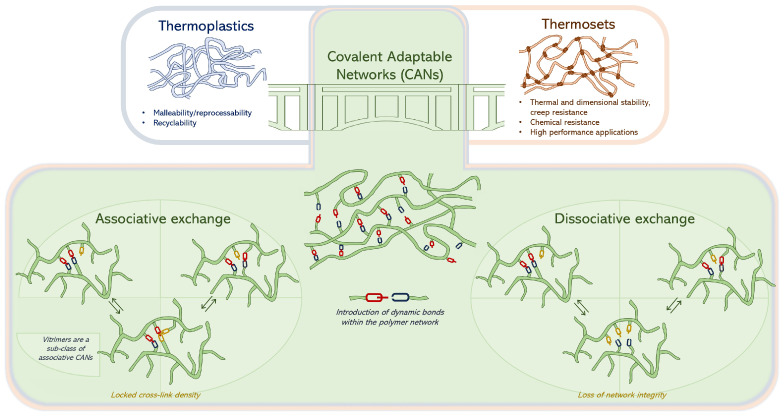
Covalent Adaptable Networks (associative and dissociative exchange mechanisms) offering the best of both thermoplastics and thermosets. Vitrimers are a sub-class of associative CANs.

Polymers are very versatile materials, used as is or as matrices in composites for various applications. They are commonly divided into two categories, thermoplastics and thermosets. While thermoplastics can be easily melted, processed and reshaped, thermosets, once cured, form irreversible three-dimensional crosslinked networks that exhibit remarkable chemical, thermal and dimensional stability as well as creep resistance [1,2,3]. However, high performance and remarkable properties come at the price of recyclability and reshape-ability, making the sustainable end-of-life management of such materials very difficult, if not impossible. To address this issue while still benefiting from the high performance of thermosets, scientists have incorporated some chemical dynamism into polymer networks to create so-called Covalent Adaptable Networks (CANs) [1,4]. Dynamic bond exchanges in CANs can be either “dissociative” or “associative” (Figure 1).

In the first case, the covalent bond is broken under one set of conditions and reformed under another, enabling network remodeling but putting at higher risk the network’s integrity and increasing the chances of side reactions during reformation of the bonds. On the contrary, associative mechanisms involve simultaneous bond dissociation and re-association, endowing associative CANs with a constant crosslink density throughout the exchange process [5]. Besides the renowned Diels–Alder dissociative exchange reaction [6], many other adaptable network chemistries have been investigated and are described in the literature [7,8]. Vitrimers are a revolutionary subclass of associative CANs discovered in 2011 by Leibler et al. [9]. They can be considered the next generation of polymer networks because they combine the malleability and recyclability of thermoplastics with the insolubility of thermosets [10,11,12,13], though they can experience more creep than traditional thermosets [14,15,16]. Similarly to other CANs, numerous exchange chemistries exist for vitrimers, such as transesterification, transamination, disulfide exchange, boronic ester exchange and many others [17,18]. These exchange chemistries can be implemented in different types of materials such as epoxies [19,20], polyurethanes [21], elastomers and rubbers [22,23,24], polyolefins [25] and benzoxazines [26,27].

**Figure 2:**
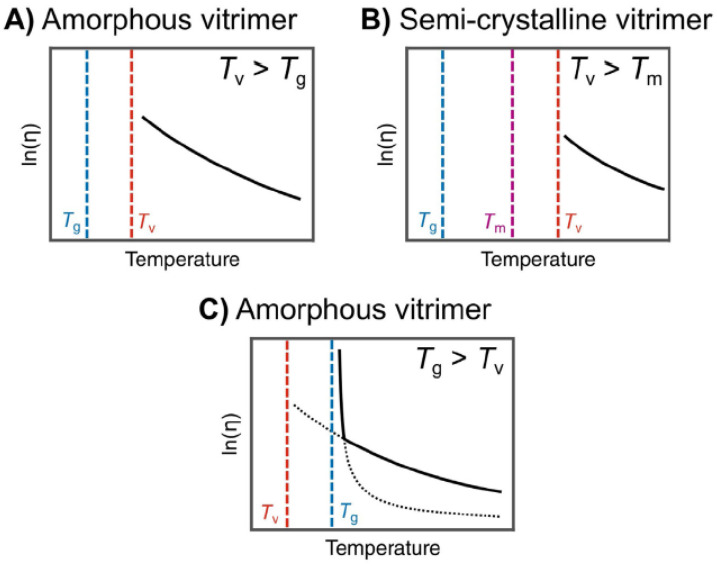
(**A**) Plot of ln(η) versus temperature of an amorphous vitrimer in which the topology freezing transition temperature, $T_{v}$, is greater than the glass transition temperature, $T_{g}$. (**B**) Plot of ln(η) versus temperature of a semi-crystalline vitrimer in which $T_{v}$ is greater than the melting temperature, $T_{m}$. (**C**) Plot of ln(η) versus the temperature for a vitrimer that features a $T_{g}$ that is greater than its $T_{\nu }$. Reprinted with permission from van Zee et al. [31].

Furthermore, in addition to the usual glass transition temperature ($T_{g}$), vitrimers have been proposed to possess a second transition temperature known as the topology freezing transition temperature ($T_{\nu }$). The unique bond exchange mechanism [17], with its well-controlled exchange rate, creates dynamic crosslinks, with consequences for the mechanical response. In this context, the position of the $T_{\nu }$ in comparison with the $T_{g}$ may be used to classify the thermomechanical behavior of the material. When a vitrimer is heated above the $T_{g}$, it undergoes a transition from a glassy to rubbery state. Further heating will increase the bond exchange kinetics, thus reducing the lifetime [28,29] of the dynamic bonds. Within the temperature range between the $T_{g}$ and $T_{\nu }$, the material behaves as an elastomer due to slow exchange reactions and the network topology remaining frozen. When the temperature reaches the $T_{\nu }$, the exchange reactions become fast enough to allow for permanent deformation. At this point, the amorphous vitrimer starts to behave as a viscoelastic liquid [30], with a melt viscosity, η, that gradually decreases following Arrhenius behavior (Figure 2A). For a semi-crystalline vitrimer with a melting temperature ($T_{m}$) lower than $T_{\nu }$, the material behaves like an elastomer between $T_{m}$ and $T_{\nu }$ (Figure 2B). Above $T_{\nu }$, it behaves like a viscoelastic liquid. Furthermore, the $T_{\nu }$ of the vitrimer can be lower than the $T_{g}$, causing the material to behave like a viscoelastic liquid at high temperatures, with a melt viscosity, η, following Arrhenius behavior.

In this case, as the temperature decreases and approaches the $T_{g}$, the degenerate reactions remain fast, and the long-range segmental motions of the polymers become the limiting factor for the topological rearrangement of the network. Here, the temperature dependence of the melt viscosity follows the Williams–Landel–Ferry (WLF) equation [17,32]. When the temperature reaches the $T_{g}$, the suppression of long-range molecular motion induces a transition from a viscoelastic liquid to an elastic solid, and the exchangeable dynamic crosslinks are trapped in the glassy matrix. Such behavior takes place in both amorphous and semi-crystalline vitrimers [31].

The topology freezing transition temperature ($T_{\nu }$) is an abstract concept with no less than three definitions reported in the literature, and based on a range of experimental approaches [33]. Values of $T_{v}$ are typically measured via dilatometry [17] or stress (σ) relaxation measurements, the latter having become the characterization technique of reference for vitrimers as well as creep experiments [34]. In their seminal publication on vitrimers, Leibler et al. defined $T_{\nu }$ as the temperature at which a viscosity of $10^{12}$ Pa·s is observed when 5% strain is applied to the vitrimer [35]. Later on, in a study of epoxy vitrimers using time-resolved multi-frequency small-amplitude oscillatory shear (SAOS) measurements (angular frequencies from 1 to 100 rad/s and a strain amplitude of 0.1%), the onset of dynamic bond exchange was observed to occur over a range of temperatures (up to 20 K in width) rather than as a pronounced shift at a single temperature [36]. Yet another report concerning silyl ether-based systems defined $T_{\nu }$ as the temperature below which glassy dynamics were observed in the materials’ relaxation spectrum [32]. In addition to the impact of the measurement approach, $T_{v}$ also varies as a function of the materials system, its intrinsic chemical structure, and the nature and content of the catalytic centers in the system [17].

Yan Ji et al. [37] attempted to detect the topology freezing temperature with a doping technique based on the use of solvent swelling to introduce aggregation-induced-emission (AIE) luminogens into an epoxy vitrimer of diglycidyl ether of bisphenol A (DGEBA) and adipic acid, as well as 1,5,7-triazobicyclodecen (TBD, 5 mol% to the COOH) as an external catalyst. In that system, drastic changes in the fluorescence of the doped material were observed when traversing $T_{v}$ in the form of shifts in the slope of fluorescence–temperature plots. Such variations in the shift in fluorescence intensity as a function of temperature indicate the temperature at which the topology of the material is changing. This approach removes the uncertainties associated with the selection of experimental parameters such as the application of an external force [34], the loading geometry and the magnitude of the strain rate for stress relaxation and dilatometry measurements, and the potential impact of such parameters on measured values of $T_{v}$ [37].

The transport characteristics of vitrimers have also been used as a means of assessing their behavior. Here, the ability of a volume of solvent to penetrate a vitrimer sample with a given thickness in a certain amount of time depends on the solubility coefficient *S* and the diffusion coefficient *D* [38]. One means of quantifying the extent of such a process is to measure the distance traveled by the penetrant, usually from the surface of the sample toward its core. This is made possible by the motion of the polymer chains within the material and depends on the amount of free volume accessible by the solvent molecules. The dynamics of bond exchange in vitrimers can make it easier for soluble molecular compounds to travel through the network, while the associative nature of such exchange reactions ensures that the network does not lose its integrity in the process. Experimentally, this has been demonstrated in the form of the enhanced diffusion of a fluorescent dye called bis(2,5-ditertbutylphenyl)-3,4,9,10-perylenedicarboximidea (BTBP) in vitrimers consisting of acrylate-based networks with both permanent and dynamic bonds, the latter based on boronate ester exchange. Two limiting cases were investigated in the context of the dynamic bonds, one being slow exchange and the other being fast exchange. The evolution of the diffusion coefficient (*D*) was reported as a function of $T_{g}$/$T_{0}$ with $T_{0}$ being set at 23 °C and $T_{g}$ changing depending on the crosslink density of the network. Huang et al. reported that at low $T_{g}$/$T_{0}$ values (and thus low crosslink densities), the diffusion coefficients are the same despite differences in the type of crosslinker. The slow dynamic bonds do not seem to influence the diffusion of the dye through the material because the rate at which bond exchange occurs is not high enough to differentiate the material from a permanent network. However, in the case of fast dynamic bonds, the bond exchange reactions (BERs) occur on timescales where penetrant hopping is possible, enhancing the diffusion of the penetrant as a consequence [39,40].

Shifting now to mechanical performance, a vitrimer intended for structural application should be able to resist creep [17,31] in spite of the dynamic nature of the bonds it contains and sustain mechanical loads over extended periods without permanent deformation [31]. Creep may be divided into three stages, referred to as primary, secondary and tertiary creep. For vitrimers, molecular mechanisms have been posited to provide the chain mobility associated with primary creep, network topology reorganization via dynamic bond exchange has been associated with secondary creep, and tertiary creep relates to defects that occur during network rearrangement [41]. The same report highlights the influence of the presence of an external catalyst in vitrimeric networks on creep behavior. The higher the catalyst loading, the higher the creep strain. On the contrary, at low temperatures, the aforementioned molecular mechanisms associated with primary creep remain inactive, resulting in the suppression of the process [41]. In another report from Hubbard et al., non-isothermal elongational creep experiments, along with stress relaxation and isothermal elongational creep experiments, were used to investigate the role of experimental parameters such as the catalyst concentration, temperature and applied axial force on the measurements of $T_{v}$. In the case of the studied transesterification-based vitrimer, the conclusions were that variations of the applied force can shift $T_{v}$ values up to 4 °C, while changes in the heating rate can shift it up to 43 °C. Finally, the sole variations in catalyst concentrations led to larger ranges of $T_{v}$ compared with $T_{g}$ [42].

Vitrimers also inspire interest in the field of composites, where adding reinforcing phases of various types, such as fibers and nanoparticles, creates the opportunity to tune the mechanical properties and repairability of these materials [43,44,45,46]. Shifts in the glass transition temperature have been observed in some cases as well, in addition to the improvements in repairability, shape memory and flexural strength [44]. Another advantage of using vitrimers as a matrix for composites is that valuable reinforcing phases (e.g., carbon fibers) can be recovered following the recycling of the materials [19,47,48].

Having said all this, it remains true that bond exchange in both CANs and vitrimers is challenging to investigate and to understand experimentally. In that context, the purpose of this thorough review is not to refer to different chemical strategies [31] that can be used to form CANs and vitrimers. Neither is it the goal to explore their behavior by characterization methods or through mesoscale simulations using, for instance, finite element modeling or micromechanical methods [49], nor to review different computational methods [50] to simulate such events. Rather, the focus of this review is the understanding of the unique behavior of CANs and vitrimers from a molecular perspective [51]. For this reason, we will not only review different molecular approaches to investigating the reversible crosslinks [52] but also discuss the structural [53,54], dynamic, mechanical and rheological behavior [33,55] inherited from the reversible crosslinks that can be revealed by molecular simulations.

The rest of this review is organized as follows: In Section 2, studies on CANs and vitrimers using the equilibrium molecular dynamics (MD) and non-equilibrium molecular dynamics (NEMD) methods of atomistic or coarse-grained models are discussed. In particular, we discuss work concerning amorphous CANs and vitrimer networks as well as vitrimer nanocomposites. Subsequently, in Section 3, efforts using the Monte Carlo (MC) methodology to model vitrimers are presented. In Section 4, we refer to and discuss studies that used hybrid Molecular Dynamics/Monte Carlo (MD-MC) approaches based on coarse-grained models. Finally, in Section 5, the conclusions of this review are summarized.

## 2. Molecular Dynamics

### 2.1. Atomistic Modeling of CANs and Vitrimers

In this section, we discuss studies that focus on CANs and vitrimer behavior by molecular dynamics simulation, using atomistic models. While vitrimers are associative CANs, their dissociative counterparts also exist, and fall under the broader umbrella of CANs as well. It is worth noting that a recent review falsely classifies all such networks as vitrimers [50]. The networks that have been investigated in the literature, by atomistic simulation, are depicted in Table 1, and consist of both CANs and vitrimers. There are various methods that have been used to simulate the crosslinks of epoxy networks by molecular dynamics simulations.

In particular, earlier methods were focused on the “cut-off” distance, where the probability of reactive sites to react was dependent on their relative distance without considering the reaction pathway [56,57]. An atomistic molecular dynamics study of BERs, using the atomistic polymer consistent force field (PCFF), was implemented to study a crosslinking CAN formed by reacting DGEBA with the crosslinking agent tricarballylic acid (at a 3:2 molar ratio) (using, in total, 5430 atoms in the simulation box) [58]. The BER was a transesterification reaction. In those atomistic MD simulations, a distance-based reaction cut-off was implemented, which accelerated the chemical dynamics [58]. In particular, bonds were created based on the proximity of reacting atoms, and the topology was accepted based on the energy of the new bond. The atomistic simulation had to be started with a low cut-off distance in order to be stable, avoiding big changes in energies due to initial reactions, and, subsequently, the cut-off distance was increased [58]. However, this methodology does not take into account the reaction pathway.

**Table 1:** CANs and vitrimers studied by atomistic molecular dynamics, force field used and property of investigation. aCAN and dCAN correspond to associative CAN and dissociative CAN, respectively.

Resin	Crosslink Agent	Network	Force Field	Property	Refs.
DGEBA	tricarballylic acid	aCAN	PCFF	stress/strength	[58,59,60]
				$R_{ee}$, welding, creep	
Tetrafunctional	trifunctional	dCAN	PCFF	welding	[61]
furan	maleimide			interfacial strength	
DGEBAEO-2(6)	MPDBMI	dCAN	PCFF	$T_{g}$, welding, shape recovery	[62]
Polyimine	Bisphenol A	dCAN	PCFF	$T_{g}$, stress/strain	[63]
				welding	
DGEBA	AFD	vitrimer	CVFF	volume, self-healing	[64]
DGEBA	AFD, HT-100	vitrimer	PCFF	self-healing	[65]
DGEBA	adipic acid	vitrimer	ReaxFF	stress, self-healing	[66]
DGEBA	AFD	vitrimer	CVFF	volume, creep	[67]
DGEBA	fatty acid	aCAN	PCFF	decomposition	[68]
Dialdehyde	diamine	aCAN	ML FF	free-energy	[69]
Epoxides	carboxylic acid	vitrimer	PCFF	$T_{g}$	[70]
DGEBA	AFD	vitrimer	PCFF	stress	[71]
		nanocomposite		$T_{g}$ self-healing	

**Figure 3:**
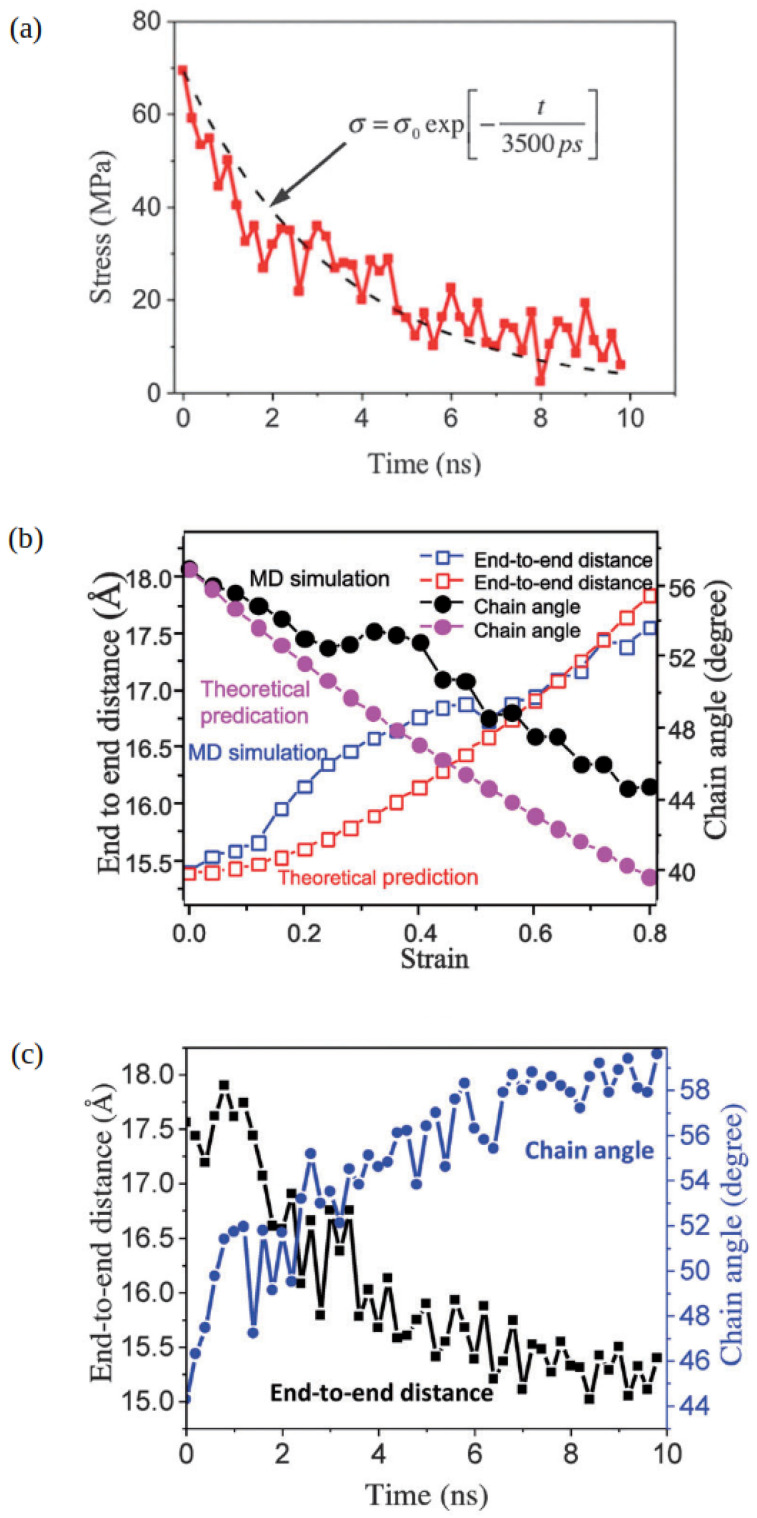
(**a**) Stress (σ) relaxation during the BER process, evolutions of the end-to-end distance ($R_{ee}$) and the average chain angle (**b**) during the uniaxial tension and (**c**) during BER iterations. Reprinted with permission from Yang et al. [58].

It was found that, even though BERs changed the macroscopic shape of the network, microscopic network characteristic features, such as the distance between two neighboring crosslink sites and the chain angle, relaxed back to the unstretched isotropic state. In particular, Figure 3a shows that the stress relaxed as the BER process continued. When the BERs slowed down, for instance, at low temperatures, the vitrimers behaved as elastomers. When the BERs were turned off, there was little stress relaxation [58]. The end-to-end distance ($R_{ee}$) and the chain angle during uniaxial deformation [72], as predicted by atomistic simulations, are depicted in Figure 3b [58] and compared with the theoretical model by Wu and van der Giessen [73,74], showing that the MD simulations follow the theoretical trend. During the BER process, Figure 3c shows that both the chain angle and the end-to-end distance recover to their original states. This means that, although the tensile deformation changes the chain orientation, the BER-induced network rearrangement returns the network to its isotropic state [58].

**Figure 4:**
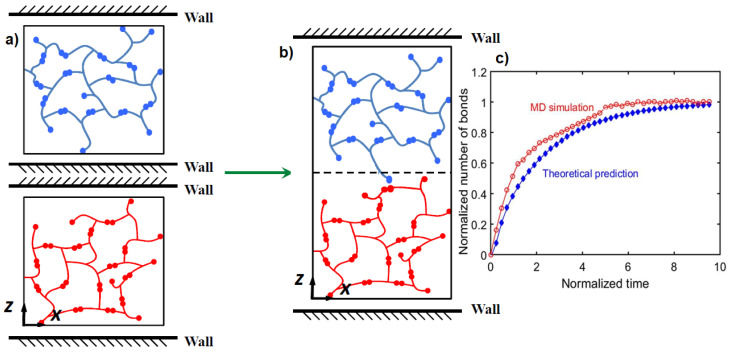
Schematic graphs of the MD simulation setup of the welding process. (**a**) The initial model with repulsive walls. (**b**) The model after the first BER occurs between the two films. (**c**) Normalized number of bonds across the interface during BER process. Reprinted with permission from Yang et al. [59].

Surface welding due to BERs of the same epoxy (DGEBA crosslinked with tricarballylic acid) was investigated by atomistic MD modeling using PCFF [59]. The initial configuration of such MD simulation employed periodic boundary conditions in only two dimensions (2D), allowing for a free surface between the reacting molecules [59] using a simulation setup which can be seen in Figure 4a,b. If the distance of the active atoms was below the defined “cut-off” distance, an active atom was connected to the existing bond. If the new bond had a lower bond energy, the BER occurred [59]. For the simulations that consider temperature effect, an additional MC step was implemented; specifically, a probability was calculated based on temperature and was compared with a random number. If the number was smaller than the probability, the BER occurred [59]. It was found that both modulus and yield strength increase with the welding time and temperature. A lower crosslink density resulted in a shorter welding time, while a low degree of polymerization lead to slower surface welding between CANs [8]. Figure 4c plots the normalized number of bonds crossing the interface in both the MD simulation and the theoretical prediction [59,75]. Here, the normalized number of bonds is calculated by using their respective saturation values, and the time is normalized by the stress relaxation time. The consistency between the two curves verifies the effectiveness of the theory of Yu et al. [59,75]. The penetration depth of a polymer chain across an interface was obtained from the MD simulations and was consistent with Stukalin’s theory [59].

When a constant stress (of 10 MPa) was applied to a network consisting of DGEBA crosslinked with tricarballylic acid and maintained at a temperature sufficient to activate the BER, a strain of 6.77% was reached before the CAN began to creep in the direction of stress, and the polymer chains aligned in the same direction. The longitudinal strain of the network in the final state was 17.3%. The aligned polymer chains resulted in an anisotropic network being mechanically stiffer in the direction of the creep, as verified by atomistic MD by Hanzon et al. [60], whereas more compliant behavior was observed in the transverse direction. In addition, the degree of network anisotropy was analogous to the amount of creep strain [60].

In another CAN system, consisting of a Diels–Alder (DA) reversible network comprised of tetrafunctional furan and trifunctional maleimide (in a 1:1 molar ratio), the surface welding was investigated using PCFF [61]. A “cut-off” distance approach was applied, where the probability of reactive sites to react was dependent on their relative distance [61]. Two separate DA networks were brought in contact, which allowed depolymerization and interdiffusion at elevated temperatures, as well as subsequent repolymerization and interfacial linkage formation at low temperatures. By using the van’t Hoff equation [61], the extent of depolymerization was correlated with temperature [61]. The welding temperature is very crucial for the welded interface. When the welding temperature is below the gel-point temperature, increasing welding time shortens the minimal path length and reduces the extensibility of the welded interface [61]. When the welding temperature is above the gel-point temperature, the interfacial strength can reach the bulk value with increasing welding time [61]. In particular, the surface welding and shape memory behaviors of CANs with thermally induced Diels–Alder (DA) reactions has also been investigated by Yang et al. by atomistic MD, using PCFF [62], using a simulation setup which is presented in Figure 4a,b. In particular, 3 networks of functionalized poly-(hydroxyaminoethers) (PHAEs) were first synthesized from two bisphenol A-type epoxy resins containing two and six oxyethylene units (DGEBAEO-2 and DGEBAEO-6) with different weight fractions [62]. The crosslinkers that were used to form the DA-induced network were 1,5-bis(maleimido)-2-methylpentane (MPDBMI) [62]. It was found that the predicted $T_{g}$ values were in good agreement with the experimental ones [62]. It was also shown that, since more DA reactions occurred, the modulus and maximum stress increased with welding time. DA networks with more flexible chains lead to lower glass transition temperatures, lower strengths and faster shape recovery [62]. The penetration depth of a polymer chain across an interface was obtained from MD simulations and compared with the theoretical predictions of Leibler [9] and Stukalin [76], revealing that the entanglement of polymer chains would reduce diffusivity [59,77,78]. BERs can improve the diffusion rate and the final penetration depth, as observed in crosslinked epoxy-polyimine networks [63].

**Figure 5:**
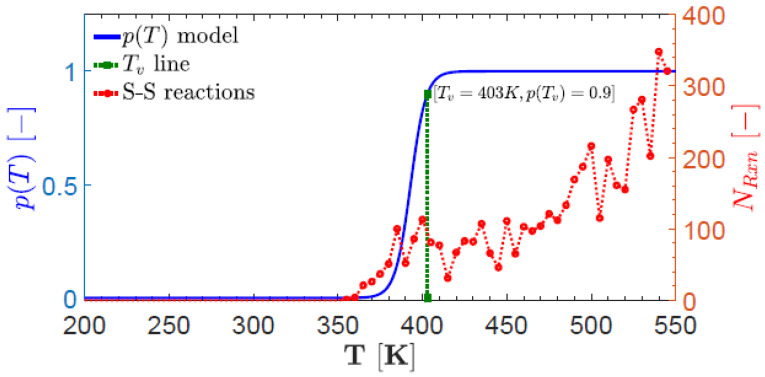
The reaction probability and the resulting number of S–S BER ($N_{Rxn}$ vs. temperature). Reprinted with permission from Singh et al. [64].

Another model vitrimer consisting of DGEBA with 4-aminophenyl disulfide (AFD) was modeled (using a stoichiometric ratio of 2:1) [64] due to its ability to achieve dynamic crosslinks in the absence of catalysts [79], using the atomistic consistent valence force field (CVFF) (using, in total, 68,608 atoms in the simulation box). In this atomistic modeling [64], an algorithm for chemical reactions was used, based on pre- and post-reaction templates with a fixed proximity cut-off distance [80], avoiding the issue of a low “cut-off” distance for stability considerations [58]. A temperature-dependent probability model was employed for the dynamic disulfide BER kinetics [81] near or above a freezing transition temperature (Equation (1)) [64], as is shown in Figure 5.

(1) $$p\left(T\right)=\frac{1}{e^{-\alpha (T-T_{\nu })}-ln\left(\frac{p(T_{\nu })}{1-p(T_{\nu })}\right)+1}$$

where $\alpha =\frac{2p(T_{\nu })}{w(1-p\left(T_{\nu }\right))}$ is determined by *w*, which is the measure of the transition from glass to rubbery state, and $p(T_{\nu })=0.9$ is the probability of the dynamic BER at $T_{\nu }$. Such approach allowed atomistic modeling of mechanical properties in vitrimers during thermal cycling above and below $T_{\nu }$ [64]. When two pairs of disulfide sites come together, they can exchange the chains attached to them. The BER could happen when sulfur atoms from different chains approach one another to within a cut-off distance of 4.12 $\overset{˚}{A}$ (double the S–S bond length [82] of 2.06 $\overset{˚}{A}$) and, when such sites were identified, the probability of the reaction was modeled as a function of temperature (Figure 5). The time-dependent deformation response of the vitrimer was predicted with and without the dynamic BERs under uniaxial stress of 500 MPa at 600 K (well above the topological transition temperature) [64].

**Figure 6:**
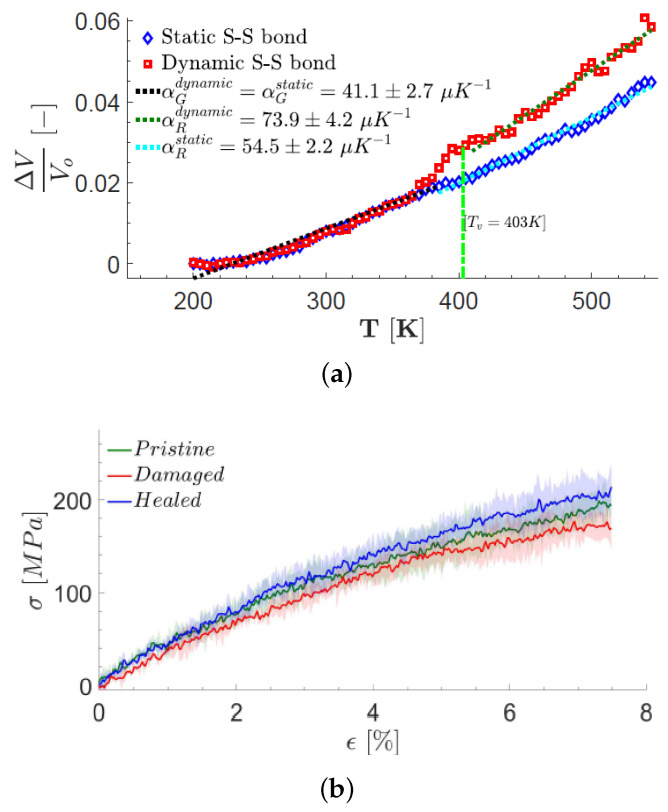
(**a**) Volumetric expansion vs. T for model with and without modeling S–S bond reactions. R and G represent the rubbery and glassy phases of the glass transition, respectively. (**b**) Stress–strain response under uniaxial tension in different directions at 300 K for damaged and healed samples. Reprinted with permission from Singh et al. [64].

When heating vitrimers above the topology freezing point, $T_{\nu }$, the dynamic BERs accelerate, which allows for the relaxation of stress applied to the vitrimers, rearranging the network topology. When the temperature decreases (cooling), the dynamic BERs slow down, and the network topology appears to be fixed on experimental timescales. Hence, they behave like permanently crosslinked networks. The V–T characteristic curve of the neat vitrimer for static and dynamic S–S bond is modeled in the simulation and shown in Figure 6a. The plot clearly shows that there is no change in the volume expansion for dynamic and static S–S bonds until the rate of bond exchange begins to increase at a temperature of 383 K, when the probability of reaction is 0.1. The dynamic S–S bond model has a higher thermal expansion coefficient than the static S–S model in the rubbery region beyond the $T_{\nu }$. This is due to the mechanical flexibility caused by the dynamic rearrangement of chains [64].

The self-healing behavior [29,76] of vitrimers, which is connected to topology and dynamics, was also modeled by atomistic molecular dynamics simulations by Singh [64] and Zheng et al. [65]. In particular, a (12,12) single-wall carbon nanotube (CNT) was inserted along the *z*-axis and allowed to displace the atoms radially in the xy plane [64]. In order to generate damage, the CNT was then pulled out of the simulation cell. The simulation cell was heated to a temperature above $T_{v}$ and the hole was healed due to dynamic S–S BERs, recovering the elastic modulus [64]. Figure 6b shows the elastic response of the damaged and healed vitrimers at 300 K. It was observed that the stress–strain response of the healed vitrimer was higher than that of the initial damaged sample in all three directions, highlighting the healing behavior. The healed structure was able to recover the pristine vitrimer elastic modulus at temperatures above the topology freezing point (Figure 6b) [64]. In addition, the atomistic model was able to show not only the healing of the vitrimer but also the complete recovery of the elastic modulus upon cooling [64]. Moreover, in another atomistic study, using PCFF, a system consisting of isocyanate-terminated poly(propylene oxide) oligomer (molecular weight ≈2000), bis(4-aminophenyl) disulfide (AFD), an isocyanate-terminated prepolymer and a tri-isocyanate crosslinker (HT-100) was modeled [65]. It was shown that the disulfide BERs enable self-healing of the polyurea [65]. As the healing time increased, the mechanical properties of the healing sample were strengthened, confirming its self-healing ability [65]. The increase in crosslink density decreased the segmental mobility, thus retarding the self-healing. Networks with shorter relaxation times gave higher healing efficiencies [65].

**Figure 7:**
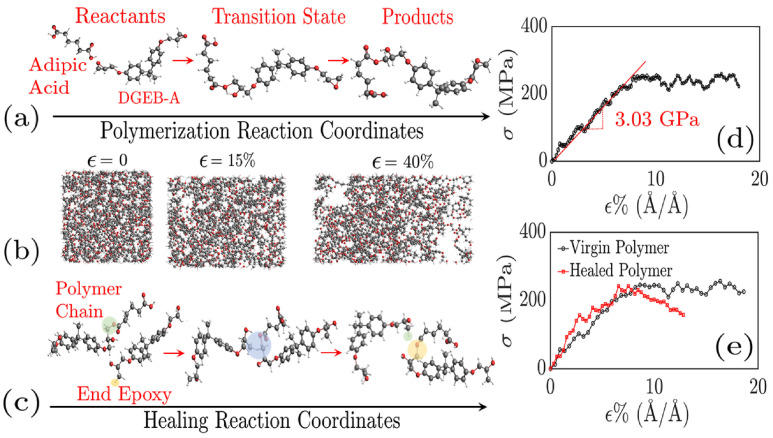
Simulating a vitrimer and its healing using molecular dynamics. (**a**) Reaction coordinates for vitrimer polymerization with DGEBA and adipic acid as initial reactants. A polymerized system was generated using an Accelerated ReaxFF algorithm and annealed to remove any local heterogeneities. (**b**) The polymerized system was virtually tested in tension at a strain rate of $\approx 10^{8}\;s^{-1}$; the snapshots at 0, 15% and 40% strain are indicated in the figure. (**c**) Reaction coordinates for the healing reaction; a polymer chain based on the reaction product of DGEBA and adipic acid undergoes a transesterification reaction, where the adipic acid switches from one epoxy molecule to the other. (**d**) Young’s modulus of the virgin vitrimer was calculated by fitting linear regression between strain values of 0–7.5% from the stress–strain curves and was found to be 3.03 GPa in the x-direction. The modulus, averaged over the x, y and z directions, was ≈2.6 GPa. (**e**) The failed specimen was healed using the Accelerated ReaxFF simulations and tensile-tested again. The healed specimen retained its modulus and strength along the x-direction. Reprinted with permission from Kamble et al. [66].

**Figure 8:**
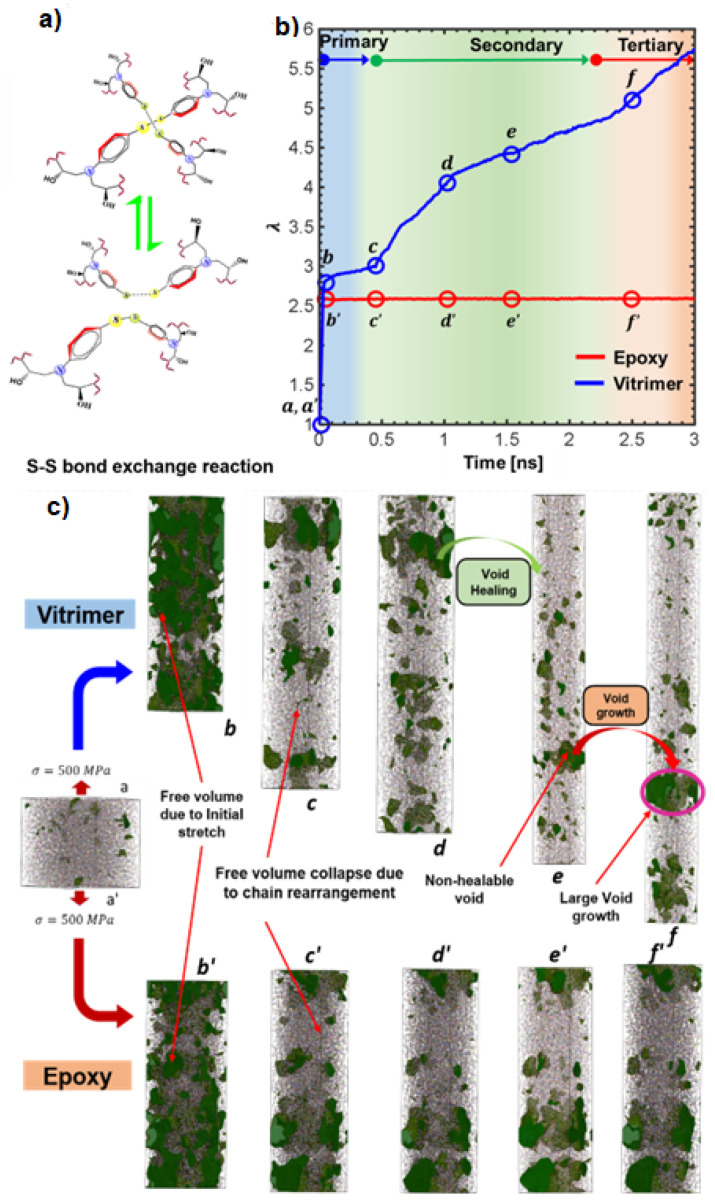
(**a**) Schematic of a disulfide BER in a vitrimeric system and (**b**) comparison of stretch ratio *l* response vs. time for vitrimers and epoxies under a constant uniaxial stress of 500 MPa at 600 K. The points from $a(a^{\prime })$ to $f(f^{\prime })$ refer to key chosen snapshots during creep deformation; (**c**) snapshots of the simulation box with free volume (green region) are shown at different time points during creep deformation. Reprinted with permission from Singh et al. [67].

The damage healing process in vitrimers [66] has also been simulated by atomistic MD using a reactive force field (ReaxFF) [83] that uses the “bond boost” approach, which was developed by Miron and Ficthorn [84] to speed up the simulations. In this approach, in addition to the cut-off distance criterion used to model epoxy networks [57,85], the reactive sites in the reactants are provided with additional energy equivalent to or slightly higher than the energy barrier for bond exchange. This method not only takes into account the reactive pathway but also enables crosslinks at low temperature, mimicking chemical reactions. This approach avoids unwanted high-temperature side reactions while still allowing for rejection of high-barrier events [85]. The vitrimer can self-heal by undergoing BER at the site of damage [66]. In that work, the vitrimer consisted of DGEBA and adipic acid in a 1:1 molar ratio [66]; using ReaxFF, and boosting only the polymerization and transesterification reactions without modeling the catalysts, the mechanical behavior and self-healing ability were investigated [66]. In particular, Young’s modulus of the virgin and healed polymers, extracted from the stress–strain curve produced by NEMD, were comparable, as is depicted in Figure 7. The calculated $T_{g}$ value of the vitrimer was in excellent agreement with the experimental measurements [66]. When the dynamic reactions are switched off, the material behaves like a conventional epoxy [67]. Atomistic MD modeling, using CVFF, was implemented in order to understand the mechanisms of creep behavior in vitrimers based on DGEBA crosslinked with 4-aminophenyl disulfide (AFD) (at a molar ratio of 2:1) (Figure 8a) [67]. In that work, the creep behavior of vitrimers was simulated by alternating loading and equilibration steps [67]. The disulfide S–S reactions were modeled using the topology-based update together with the pre- and post-reaction templates [67]. Extreme conditions in the simulations were applied in order to investigate all regimes of vitrimer creep, which is known as a slow phenomenon, whereas the timescale of MD simulations was faster [67]. A second assumption in that simulation effort was that bond rupture of crosslinked epoxy bonds was not modeled to suppress such mechanism of failure, focusing on molecular mechanisms that occurred in ambient conditions [67].

By applying tensile stress, the vitrimer shows an immediate increase in strain due to chain rearrangement, referred to as the primary creep response. In that regime, the vitrimer is more compliant than the epoxy and shows an initial increase in voids due to chain rearrangement as soon as the load is applied. A small increase in primary creep with reaction probability was observed, whereas the primary creep strain is relatively unaffected by the reaction probability [67]. Following primary creep, the epoxy does not show a significant increase in the creep strain; in contrast, the vitrimer shows an increase in strain and a decrease in stiffness along the loading axis (Figure 8b) [67]. In addition, elongation due to chain rearrangement also takes place. Very little void growth is seen, and this secondary regime, which is referred to as secondary creep, is associated with network rearrangement [41] and takes place due to dynamic exchange reactions, enabling the orientation of the bonds with respect to the loading direction. This leads to a decrease in axial stiffness, resulting in greater creep strain, during the secondary creep [67]. In that regime, the reaction probability has a strong effect on the strain rate, as was also observed in experiments [41]. A third regime, “tertiary creep” (Figure 8b), was also observed, in which void growth (Figure 8c) occurred at high reaction probability and high applied stress [67]. That tertiary behavior corresponds to systems with a high amount of catalysts [41]. In the conventional epoxy, on the other hand, only a primary creep response (driven by chain rearrangement) and a very slow secondary creep response (due to chain mobility around free volume) occurred [67]. In order to mitigate creep in vitrimers, an effective strategy would be either to include additives to prevent the initial realignment of dynamic bonds, as was observed experimentally by the addition of metal-complexes that decrease chain-to-chain interactions [86], or to add a good number of permanent bonds [13].

Furthermore, an atomistic MD study using PCFF was used to show the decomposition of CANs based on epoxy (DGEBA) cured with fatty acid monomers in an ethylene glycol solvent via transesterification-type BERs by using PCFF [68]. In that work, a larger “cut-off” distance than the one of the study by Yang [58] was used to enhance the reaction rate of BERs. Such adaptable network decomposition was controlled by the diffusion of chain segments into the solvent [68]. As the decomposition proceeded, chain segments accumulated at the polymer–solvent interface and formed a thick gel layer. In addition, the decomposition of CANs based on epoxy (DGEBA) crosslinked with a trifunctional fatty acid (in a 3:2 molar ratio) [87] in octamethylene glycol or ethylene glycol solvents via transesterification-type BERs was simulated, using PCFF, in order to study the influence of solvent size on CAN decomposition. In that atomistic modeling, the “cut-off” criterion approach was also applied [87]. The results showed that the larger octamethylene glycol molecules had less mobility, which limited the solvent diffusion into the polymer network, leading to slow decomposition behavior with a thin gel layer at the polymer–solvent interface [68]. Solvent and polymer molecules had sufficient time to diffuse when the evaporation proceeded at a controlled slow rate [87].

All previous simulations efforts were based on empirical atomistic force fields, such as CVFF and PCFF, or ReaxFF. However, very recently, a machine-learning (ML) force field (FF) based on density functional theory (DFT) ab initio simulations [50] was developed for polyimine CAN systems (specifically based on dialdehyde and diamine monomers) in order to obtain DFT-level accuracy in energy and atomic force predictions [69]. By combining the ML force field with enhanced sampling methods, including metadynamics and umbrella sampling, the free energy profiles of amine–imine exchange reactions in networks, both with and without water molecules, were calculated [69]. In particular, the ML force field described the change in chain connectivity and stress distribution induced by amine–imine exchange reactions and reproduced reaction kinetics and transition state geometries that could not be achieved by empirical force fields [69].

An MD–ML framework was also used to achieve the inverse design of bifunctional transesterification vitrimers [70]. A vitrimer dataset was created using carboxylic acids and epoxide monomers taken from the ZINC15 database [88]. The $T_{g}$ values were calculated on a subset of the dataset using MD simulations and the results obtained were calibrated using a Gaussian process (GP) regression model [70]. The GP regression model tried to predict the value $\Delta T_{g}^{GP}$ in Equation (2) [70], where $T_{g}$ was the experimental $T_{g}$ from one of 295 polymers gathered in the ZINC15 database.

(2) $$T_{g}=T_{g}^{MD}+\Delta T_{g}^{GP}$$

Here, $T_{g}^{MD}$ was the $T_{g}$ calculated by the MD scheme and $\Delta T_{g}^{GP}$ was the difference between $T_{g}$ and $T_{g}^{MD}$. The polymers in the dataset were represented using fingerprints of the repeating units. The performance reported is $R^{2}=0.85$ and has been calculated by leave-one-out cross validation (LOOCV). The dataset was then represented using dual encoders and decoders, so that the vitrimer latent space is made continuous, and similar vitrimers are organized to be positioned close to each other. This meant a flexible exploration of said latent space using a graph variational autoencoder (VAR) model [70].

**Figure 9:**
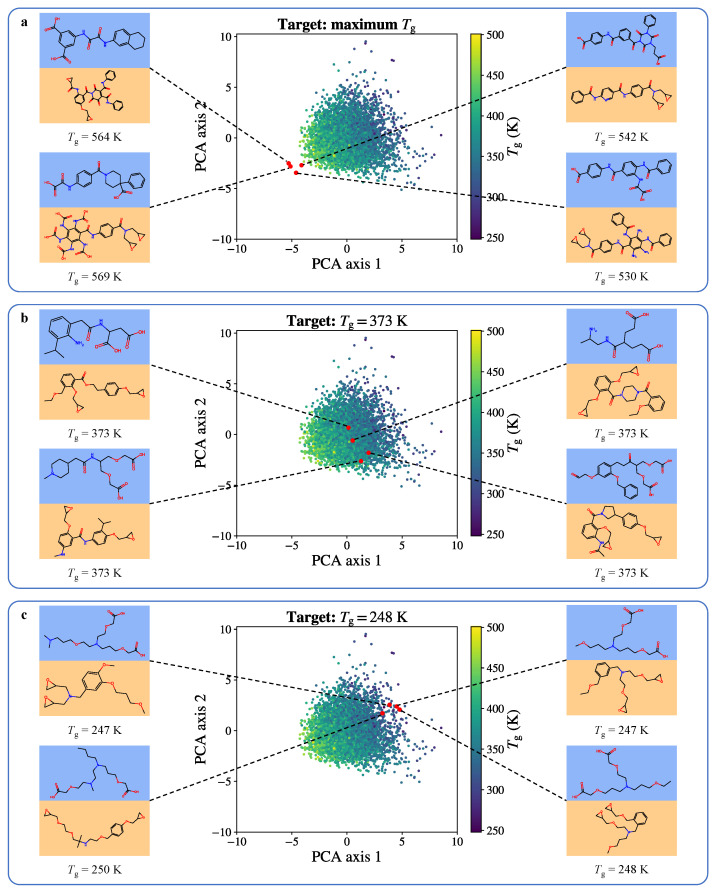
Novel vitrimers with different target $T_{g}$ values generated by inverse design based on Bayesian optimization and their locations in the latent space visualized by principal component analysis (PCA). (**a**) Maximum $T_{g}$. (**b**) Target $T_{g}$ = 373 K. (**c**) Target $T_{g}$ = 248 K. All presented $T_{g}$ values of proposed vitrimers are validated by MD simulations and GP calibration. Reprinted with permission from Zheng et al. [70].

The authors reported decent accuracy and efficiency for the framework. Novel vitrimers were then generated using different $T_{g}$ targets, both within and beyond the training distribution [70]. The vitrimer candidates for specific $T_{g}$ targets have been reported to occupy a small region of the latent space, as well as specific molecular descriptors, which match our current structure—$T_{g}$ relationship knowledge. The proposed framework seemed generic enough to be expanded to other properties or types of polymers [70]. Inverse design was achieved using the variational autoencoders (VAEs) and its property predictor ($T_{g}$). Latent vectors are optimized, decoded into vitrimers, then recoded as predicted $T_{g}$ values. This objective was implemented in a Bayesian Optimization (BO) workflow in order to avoid reproposition of the same vitrimer. The 100 candidates closest to the target were chosen for MD calculation and GP calibration of their $T_{g}$ values. Novel vitrimers were thus generated using different $T_{g}$ targets, both within and beyond the training distribution, as shown in Figure 9 [70]. It was noted that, although the Gaussian process calibration of the calculated $T_{g}$ values produces decent agreement with experimental $T_{g}$ values, the authors report using a system size of about 4000 atoms and a cooling rate of 50 ps per 10 K temperature step, which represented a relatively small size and fast rate, respectively, compared with the bulk of studies presented in this review.

### 2.2. Atomistic Modeling of Vitrimer Nanocomposites

**Figure 10:**
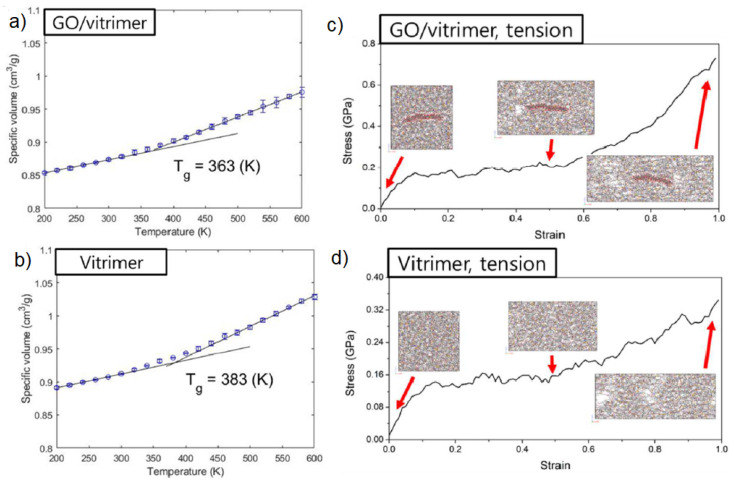
Specific volume vs. temperature graph of (**a**) GO/vitrimer and (**b**) vitrimer systems. Calculated $T_{g}^{\prime }$s of GO/vitrimer and vitrimer are 363 K and 383 K, respectively. Stress–strain curves and snapshots at engineering strains of 0, 0.5 and 1.0 for (**c**) GO/vitrimer, tension, (**d**) vitrimer, tension. Reprinted with permission from Park et al. [71].

The incorporation of nanofillers inside the vitrimer matrix can result in reinforcement of the vitrimeric matrix, leading to an improvement in mechanical, thermal and/or other properties. From an atomistic simulation point of view, there is only one effort which addressed the behavior of a vitrimer nanocomposite. In particular, a graphene oxide (GO) vitrimer nanocomposite composed of DGEBA and 2-aminophenyl disulfide (AFD) hardeners was investigated by atomistic molecular dynamics, using the PCFF [71]. In that work, an algorithm was developed that could break and recreate disulfide bonds repeatedly during the simulation [71]. It was shown that GO reduced the vitrimer $T_{g}$, as seen in Figure 10a,b, from 383 to 363 K. In addition, self-healing [29] behavior took place at temperatures above $T_{g}$. The vitrimer nanocomposites self-heal better than the pure vitrimers, accompanied by an increase in the dynamic bond exchange rate thanks to the addition of GO nanofiller, which is attributed to the lower $T_{g}$ of the nanocomposites [44,71]. This result was also observed experimentally, with the addition of GO into a disulfide-type vitrimer, by Krishnakumar et al. [44,89]. As can be seen in Figure 10c,d, the hardening slope steepens at a strain level above 0.6. The nanocomposites self-heal better than the pure vitrimers, since they self-heal completely. The addition of GO nanofiller stimulates the BER and was found to induce a consistent increase in the number of new disulfide bonds during the self-healing process [71].

### 2.3. Coarse-Grained Modeling of CANs and Vitrimers

**Figure 11:**
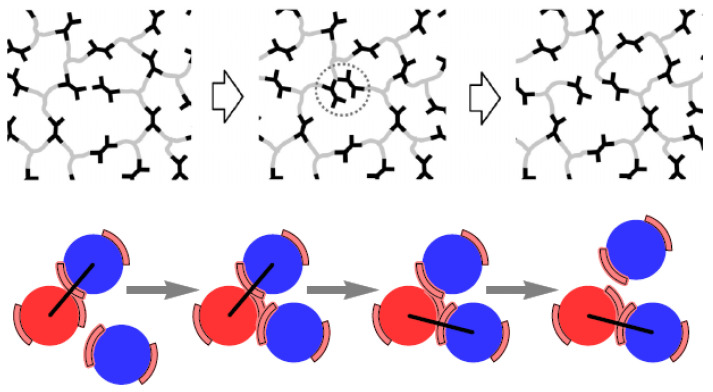
Schematic of the bond-switching mechanism in vitrimers (**top**) corresponding to the patchy particle model (**bottom**). In both cases, network reorganization can take place by a bonding site switching from its current bonding partner to another nearby free interaction site of the right species. Note that bonds are only possible between particles of different types. Reprinted with permission from Smallenburg et al. [90].

In this section, we discuss studies that focus on vitrimer behavior by molecular dynamics simulation using coarse-grained models. In particular, a patchy model (Kern–Frenkel model) for vitrimers was developed by Smallenburg et al. [90,91], depicted in Figure 11, to study phase behavior and vitrimer dynamics under good solvent conditions. The free energy of this model, limited to strong (chemical) bonds between the particles, was studied by both computer simulations (event-driven molecular dynamics) [90] and the Wertheim thermodynamic perturbation theory [92,93]. By reproducing the bond-switching mechanism using this simple model system, the model captured the strong glass-forming ability of vitrimers. Theories developed for bond association in polymers were tested for vitrimers and showed that a non-Arrhenius behavior reflects the strong decoupling of the bond exchange barrier crossing event with segmental and α–relaxation, as well as bond breaking and dissociation [94,95]. At low densities, phase separation of the vitrimers was observed in the presence of a solvent. This phase separation is analogous to the gas–liquid phase separation observed in low valence colloids, revealing new insights concerning swelling behavior in solvents, though the patchy particle model does not explicitly consider solvents [90].

In another work, a coarse-grained model was used to study the dynamics of star polymer networks, such as the eight-arm star polymer, which terminates with a reactive site [96] induced by dynamic bond exchanges [96,97,98]. The coarse-grained star polymer is a sequence of beads and strings in which beads interact through the pure repulsive Weeks Chandler Potential (WCA) (Equation (3)) [96].

(3) $$V_{WCA}=4\epsilon_{d}\left[{\left(\frac{\sigma_{d}}{r_{ij}}\right)}^{12}-{\left(\frac{\sigma_{d}}{r_{ij}}\right)}^{6}+\frac{1}{4}\right]$$

up to a “cut-off” $r_{cut-off}=2^{1/6}\sigma_{d}$, where $\sigma_{d}$ is the diameter of the particle.

**Figure 12:**
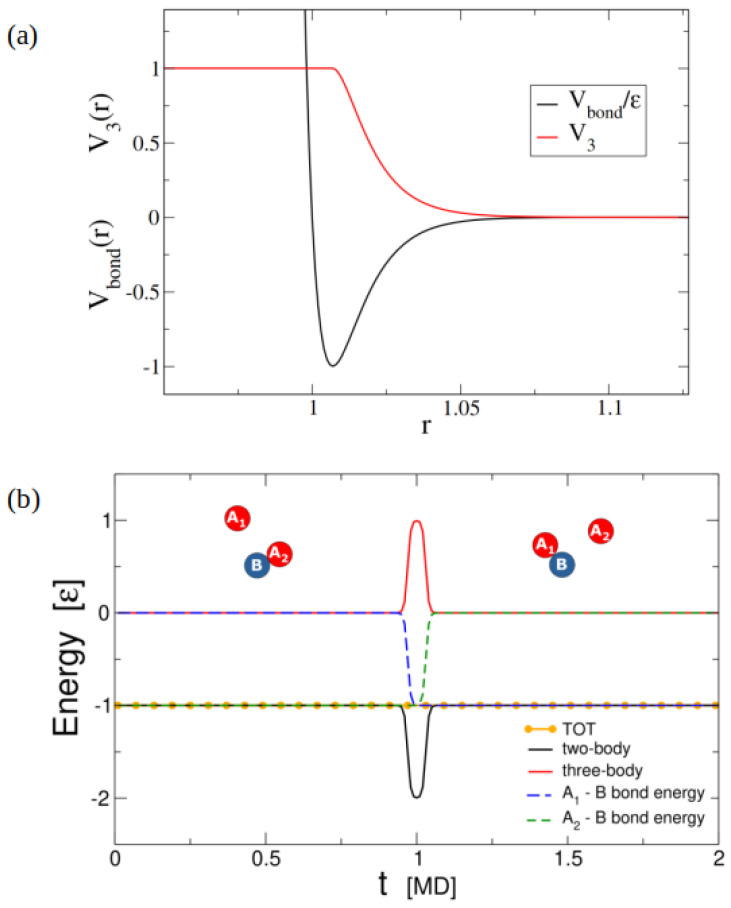
(**a**) Radial dependence of a typical (normalized) bond potential ($V_{bond}\left(r\right)/\epsilon_{LJ}$) and of the associated $V_{3}\left(r\right)$ potential. For $r>r_{min}$, the two potentials are opposite $V_{bond}\left(r\right)/\epsilon_{LJ}=-V_{3}\left(r\right)$. $V_{3}\left(r\right)$ is dimensionless, as is $V_{bond}\left(r\right)/\epsilon_{LJ}$. $\epsilon_{LJ}$ has reduced energy units and *r* has its units reduced by $\sigma_{LJ}$. Reprinted with permission from Sciortino [99]. (**b**) Time dependence of different components of the energy along a swap event at t = 1. When the particle A1 gets closer to B, its two-body energy decreases (blue dashed line), but this change is compensated for by the three-body term (red). The triplet state is short-lived; in fact, particle A2 leaves quickly after the formation of the triplet. Noticeably, the total energy (yellow) stays constant. The energy has its units reduced by $\epsilon_{LJ}$, and *t* has its units reduced by $\sigma_{LJ}\sqrt{m/\epsilon_{LJ}}$. Reprinted with permission from Ciarella et al. [98].

To model swappable covalent bonds, an algorithm was developed by Sciortino [99] based on a system composed of two types of particles (A and B), each having a number of interacting sites providing the particles with functionality. The algorithm should fulfill two conditions: (i) neither site should be able to form more than two bonds, and (ii) the activation energy of the swap process may be tuned from infinity (no swap processes, only bond breaking and re-forming) to zero (no energetic cost for swapping) [99,100]. In order to fulfill the above conditions, a combination of two-body and three-body interactions [96,99] is needed. The two-body potential is a Lennard–Jones (LJ) potential (Equation (4)) acting only between reactive sites:

(4) $$V_{bond}=4\epsilon_{d}\left[{\left(\frac{\sigma_{d}}{r_{ij}}\right)}^{20}-{\left(\frac{\sigma_{d}}{r_{ij}}\right)}^{10}\right]$$

where $\sigma_{d}=5$ $\overset{˚}{A}$ and $\epsilon_{d}=4$$k_{B}T$ up to a distance of $r_{cut-off}=2.5$$\sigma_{d}$. An additional repulsive three-body potential (Equation (5)) makes it possible to control the energetic barrier for swapping, going from the non-swapping case to the freely swapping one [99]. In particular, the three-body potential (given by Equation (5)) acts between all triplet bond sites (ABA, BAB) [100] (Figure 12) and can be used to simulate bond swaps of CANs, using coarse-grained models, in standard molecular dynamics in order to mimic exchange reactions [96,99]. However, to design a proper three-body or multi-body potential for a multivalent network system remains challenging. In addition, the three-body potential method may misbehave at high densities, where interactions with more than two particles are frequent [101].

(5) $$V_{three-body}=\lambda \sum_{ijk}\epsilon_{LJ}V_{3}\left(r_{ij}\right)V_{3}\left(r_{ik}\right)$$

The sum of Equation (5) runs over all triplets of bonded particles (particle *i* bonded with both *k* and *j*). $r_{ij}$ is the distance between particle *i* and *j*, and λ is a parameter that can be tuned to interpolate between the limits of swapping (λ = 1) and non-swapping (λ >> 1) bonds. The pair potential $V_{3}\left(r\right)$ is defined as

(6) $$V_{3}\left(r\right)=-\frac{V_{bond}\left(r\right)}{\epsilon_{LJ}}$$

for $r_{min}\;\leq \;r\;\leq \;r_{cut-off}$, whereas it takes the value of 1 for $r\;\leq \;r_{min}$. We can see the profile of $V_{bond}$ and $V_{3}$ in Figure 12a. In Figure 12b, we can see the temporal evolution of energy during a swap event.

As $A_{1}$ gets closer to *B*, its two-body energy ($V_{bond}$) decreases, but its contribution ($V_{3}\left(r_{A_{1}-B}\right)$) to the three-body term increases simultaneously. Thus, the overall two-body energy decreases. After $A_{1}$ reaches $r_{min}$, $A_{2}$ will move away from *B*, increasing its two-body energy ($V_{bond}$) but decreasing its contribution ($V_{3}\left(r_{A_{2}-B}\right)$) to the three-body term. The overall energy does not change throughout the swap. There is a short time when the triplet state exists.

$V_{three-body}$ is a repulsive potential whose amplitude can be controlled by the choice of parameter λ. The three-body energy parameter λ directly controls the swap rate by dictating the energy required for a swap to take place, thus mimicking parameter control swap frequency (for instance, catalyst concentration [100]). In practice, the three-body potential turns bond-swap events into a continuous process: free binding sites approach an existing bond via a tunable barrier, and, after the exchange, the unbound partner leaves via the same pathway [96]. This approach has an advantage over the MC approach in that the dependence of the exchange probability on physical parameters such as the bond force arises naturally and does not have to be added manually into an acceptance criterion [96]. This approach, originally used by Sciortino [99], can be applied to any network materials featuring chemical moieties that associatively swap, such as vitrimers. In addition, bond swapping can speed up simulations of materials containing strong bonds that can otherwise become trapped in metastable states. The methodology of applying three-bond potentials is suited to molecular dynamics simulations and allows the dynamics of such network materials to be studied [98]. In addition, such algorithms can intrinsically capture the physical effects of parameters affecting the swap rates, such as the mass of the swapping moieties, which would have to be added by hand in a methodology that involves topology-altering MC steps [98].

**Figure 13:**
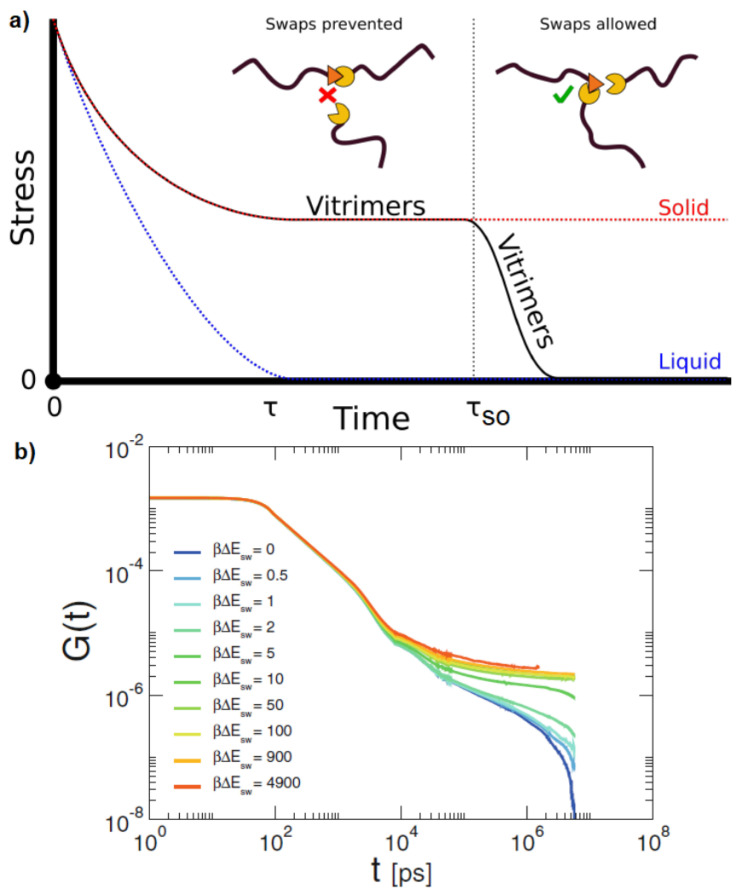
(**a**) Schematic of stress relaxation of vitrimers. Up to $t\;\approx \;\tau_{so}$ swaps are prevented, so vitrimers behave like viscoelastic solids and part of the stress is retained. After the activation of the swap reaction, stress can be relaxed through swaps, producing a liquid-like behavior. Reprinted with permission from Ciarella [106]. (**b**) Stress relaxation for the vitrimeric star network, for a range of swap barrier values. After a regime of quick relaxation due to chain rearrangement, a solid plateau is approached. For energy barriers lower than $\beta \Delta E_{sw}=10$, swap rearrangements trigger a second relaxation. G(t) is in MPa units. Reprinted with permission from Ciarella et al. [96].

A schematic stress relaxation process is tied to the bond swaps, shown in Figure 13, where no swaps take place up to $t\;\approx \;\tau_{so}$ (which is the time for a solid network to reach its elastic plateau), meaning that vitrimers have a solid-liquid-like behavior. At shorter times than $\tau_{so}$, the stress relaxation is dominated by the Rouse mode of chains [102]. When swaps are allowed, vitrimers can relax stress and behave like a liquid over long timescales. Simulations by Ciarrella et al. show that the activation mechanism allows the network to reconfigure and explore distinct topological configurations, with exchange reactions mediating and speeding up the stress relaxation (Figure 13) [96]. Defects in the model vitrimer facilitate the bond exchange, thus accelerating stress relaxation in the network [96]. The stress relaxation modulus G(t) is equal to the shear-stress autocorrelation function $C_{\tau }\left(t\right)$ in the liquid phase. However, in the solid phase, G(t) follows Equation (7) [96]:

(7) $$G\left(t\right)=C_{\tau }\left(t\right)+G_{eq}-C_{\tau \infty }$$

where $G_{eq}$ is the shear modulus and $C_{\tau \infty }$ is the long time limit of $C_{\tau }\left(t\right)$ [96,103]. G(t) can also be computed directly, by MD simulations, using the stress tensor $\sigma_{ab}$ with Equation (8) [52]:

(8) $$G\left(t\right)\approx C_{\tau }\left(t\right)=\frac{V}{k_{B}T}<\bar{\sigma_{\alpha \beta }\left(t\right)\sigma_{\alpha \beta }\left(0\right)}>$$

where the off-diagonal components of the stress tensor $\sigma_{\alpha \beta }$ are evaluated in the NVT ensemble at constant number of molecules *N*, volume *V* and temperature *T*. The bar and brackets denote averaging over time and ensemble, respectively. According to Figure 13, the time needed for a solid network to reach its elastic regime is ≈500 ns. At shorter times, the stress relaxation is dominated by the Rouse modes of polymer chains [102]. For energy barriers higher than $\beta \Delta E_{sw}=50$, where $\beta =1/k_{B}T$, and $\Delta E_{sw}$ is the energy barrier for the bond swap, the topology remains fixed, and the plateau extends beyond times that can be reached by simulations [96]. For a low energy barrier for the swap move (smaller than $\beta \Delta E_{sw}=10$), the network rearranges itself through bond swap moves and a second relaxation is observed, which is the hallmark of transient networks [76,90,103,104]. The adhesion of two vitrimer samples (made from star polymers) was also studied, and they were found to bond together on timescales much shorter than the stress relaxation time [105]. The swap mechanism allows the star polymers to interdiffuse through coordinated swap events. In addition, a non-stoichiometric network with a mixture of tetrafunctional and bifunctional (in excess) particles that incorporate the bond-swapping mechanism was investigated [100]. The swapping mechanism is initiated when the thermal vibration of the network brings an unreacted site near to an existing bond, and a swap process takes place that allows for network restructuring [100]. Two distinct relaxations were observed at low wavevectors: (i) a fast vibrational damped mode, and (ii) a slow process associated with restructuring of the network [100].

The healing process is much faster and not dependent on the star polymer mobility, but rather on crosslink swaps [65,105], which are more frequent when the crosslink density is high [105]. For star polymers, healing is driven by the fluctuation of single arms. Stress relaxation requires many swap events involving all arms on each star polymer [105]. The MD simulation setup corresponded to a realistic physical scenario in which the bond exchange kinetics of the reversible crosslinkers are slow; that is, the average lifetime for a reversible crosslink to remain attached to its two partners is far longer than the (microscopic) simulation timescale [107,108]. In experiments, the reversible crosslink bonding half-life spans times on the order of milliseconds to seconds, clearly far longer than the molecular timescale being examined in the MD simulations [109]. By adapting the bulk density, the fragility of vitrimers at $T_{g}$ can be adjusted, revealing fragile (super-Arrhenius), robust (Arrhenius) and exceptionally resilient (sub-Arrhenius) behaviors [105]. A Gaussian chain theory and polymer self-consistent field theory for networks were developed to construct a microscopic picture for how reversible crosslinks can toughen a polymer network without affecting its elasticity [107]. Maximizing of polymer entropy can drive the reversible crosslinks to bind preferentially near the permanent crosslinks in the network, leading to local molecular reinforcement. The network is thereby globally toughened, while the linear elasticity remains largely unaltered [107].

**Figure 14:**
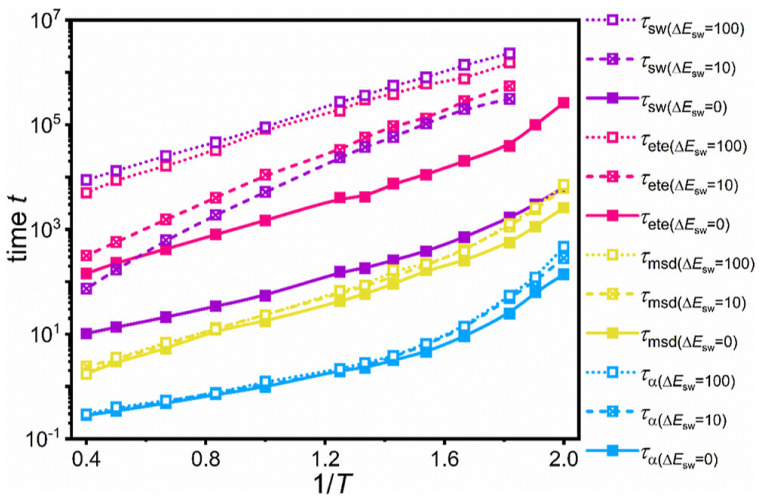
Temperature and energy barrier dependence of the characteristic times of multiscale dynamics. $\tau_{sw}$ represents the bond swap dynamics and is obtained using the bond autocorrelation function $C_{sw}$. $\tau_{ete}$ represents the polymer chain relaxation dynamics and is obtained using the end-to-end vector autocorrelation function $C_{ete}$. $\tau_{msd}$ represents the bead mobility and is obtained using the mean square displacement of the polymer beads $g_{1}$. $\tau_{\alpha }$ represents the chain segmental relaxation dynamics and is obtained using a two-step relaxation function $F_{s}\left(q_{0},t\right)$. *t* is in reduced units, having its units reduced by $\sigma_{LJ}\sqrt{m/\epsilon_{LJ}}$, and *T* is in reduced units, having its units reduced by $\epsilon_{LJ}/k_{B}$. Reprinted with permission from Zhao et al. [112].

In another study of vitrimers using coarse-grained models and dynamic covalent bond exchange of reactive beads simulated by the three-body potential [99] (using Equation (4) for reactive sites), it was found that the BER lifetime follows an Arrhenius relationship with energy barrier swap ($\Delta E_{sw}$) and temperature [110]. However, when modeling the bond exchange with a nonbonded Lennard–Jones potential, it is greatly influenced by thermal fluctuations or external forces [111]. Moreover, using the same model, the time–temperature superposition (TTSP) and time–energy barrier superposition (TESP) principles were investigated since both the bond swap energy barrier and the temperature were reported to have an effect on multiscale dynamics [112]. The dynamics involved were segmental dynamics, polymer bead mobility, polymer chain dynamics and bond swap dynamics. TTSP was valid for polymer bead mobility and bond swap dynamics, and was valid for segmental dynamics and polymer chain dynamics only at low temperatures and low energy barriers, respectively [112]. TESP was only valid for segmental dynamics [112]. Moreover, the scale of the different dynamical behaviors revealed that segmental dynamics were the fastest, followed by polymer bead mobility and polymer chain dynamics; bond swap dynamics were the slowest [112]. However, their dependence on temperature and energy barrier were nonequivalent, as shown by their characteristic times in Figure 14. The chain segmental relaxation dynamics and the bead mobility had a weak dependence on energy barrier, while the polymer chain relaxation dynamics and the bond swap dynamics were significantly slower, with an increase in energy barrier [112]. Due to the different timescales of those dynamics, it was possible to tune the properties of a vitrimer by isolating a certain scale. For example, if the bond exchange scale and chain relaxation scale were separated (by tuning the energy barrier), then the bond exchange (which affects dynamic reversibility) and the chain relaxation (which affects material stability) could be tuned separately to achieve controllable self-healing properties in the vitrimer [112].

As in glass-forming materials, the chain segment relaxation was characterized by two regimes, which may be described by the Vogel–Fulcher–Tammann (VFT) equation at low temperatures and the Arrhenius equation at high temperatures [95,112]. Due to the loss of free volume of the segments with increasing temperature, the VFT equation predicted a rise in the relaxation time of the polymer chain segments [112]. Meanwhile, the Arrhenius equation predicted an exponential decrease with increasing temperature since the thermal energy was enough to overcome the activation energy barrier to segmental motion [112]. It was shown that TTSP holds near the critical transition temperature, but as temperature increases, relaxation accelerates, and the material dynamics become homogeneous, causing the failure of the TTSP. TESP fails because of the complex influence of energy barriers on material dynamics [112]. In addition, there was investigation of the effect of $\Delta E_{sw}$ on the mechanical behavior of vitrimers using NEMD [110]. It was found that there is an optimal $\Delta E_{sw}=100$ intermediate value to maximize mechanical performance, since overly fast BERs led to a reduction in chain orientation, and overly low BERs led to bond breaking [110]. The self-healing capability was high with $\Delta E_{sw}=10$, and increasing the self-healing time and temperature enabled the vitrimeric material to exhibit a high degree of self-healing [110].

The viscoelasticity [113] of dynamic covalent polymers was also studied by tuning dynamic covalent bond concentration and the temperature [114]. The storage and loss moduli were increased with increasing numbers of dynamic covalent bonds, but the storage modulus decreased drastically at the critical shear strain amplitude $\gamma_{0}$ at the same point as the loss modulus [114]. The two main factors contributing to the drop in storage modulus were the orientation and deformation of chains, as well as the exchange of the dynamic covalent bonds at the critical shear strain amplitude $\gamma_{0}$. The bond dissociation energy per dynamic covalent bond and lifetime were also increased with dynamic covalent bond content at strains below the critical value $\gamma_{0}$, but the contrary was observed for strains higher than the critical value $\gamma_{0}$, reflecting exchange within the dynamic network [114]. Moreover, the storage and loss moduli were decreased at high temperatures since the lifetimes were decreased, while the bond energy per dynamic covalent bond was increased at high temperatures [114]. Curiously, the loss modulus maximum peak value at the critical shear strain amplitude vanished when reducing temperature, an observation attributed to slow exchange dynamics. The self-healing process was also simulated quantitatively, and the characteristic self-healing time also shows an Arrhenius dependence on temperature [114].

### 2.4. Coarse-Grained Modeling on Vitrimer Nanocomposites

A simulation work on vitrimer nanocomposites was implemented, modeled by MD and NEMD (uniaxial tensile deformation) using coarse-grained model, and contained unentangled (with *N* = 60 monomers) vitrimers and spherocylinder nanorods [115] (of 10% volume fraction) [116]. An Arrhenius relation was observed between the BERs, the energy barrier and the characteristic time (τ) of BER. By using such coarse-grained model, the BER was not greatly affected by the volume fraction of nanorods. However, the interfacial interaction between nanorods and polymers had a significant impact on the relaxation mode of BERs and nanorod orientation relaxation [116]. In particular, an increase in interfacial interaction slowed down the BER process, even at low bond exchange energy barriers, during uniaxial stretching and stress relaxation processes [116]. The mesh size of the vitrimer nanorod composites was comparable to that of the nanorods introduced. This implied that any variations in the mesh size affected the orientation dynamics of the nanorods [116]. A high nanorod aspect ratio helped to achieve high alignment during uniaxial stretching. The results demonstrated that the nanocomposite systems that retain a higher nanorod orientation exhibited a superior modulus and mechanical strength [116]. Another recent simulation effort containing unentangled (with N=60 monomers) vitrimers and spherical nanoparticles was implemented by MD using coarse-grained models [117]. It was shown be independent of BERs in the vitrimer matrix and interface [117]. By varying the $\Delta E_{sw}$ in the matrix and interface, it was shown that an appropriate $\Delta E_{sw}$ value is beneficial not only to ensure enough BERs to homogenize the fracture region but also to obtain a certain network strength in achieving a high dissipation work [117].

## 3. Monte Carlo

**Figure 15:**
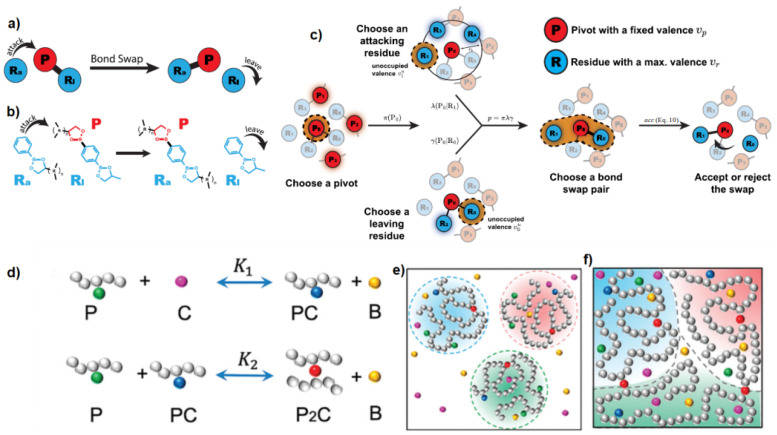
A general bond swapping algorithm. (**a**) A pivot P is attacked by a residue $R_{a}$ and forms a new bond while breaking an existing bond and releasing a leaving residue $R_{l}$. The bond is swapped from $R_{l}$ to $R_{a}$ while the total valences are conserved throughout the process. (**b**) A typical chemical realization of the model in a vitrimer undergoing dioxaborolane metathesis. (**c**) Schematic of the bond swap algorithm. The algorithm first chooses a random pivot ($P_{0}$) from the sampling space with the probability $\pi (P_{0})$. Then, it searches for all residues with vacant valences within $r_{b}$ and randomly selects one as the attacking residue ($R_{1}$) with the probability $\lambda (R_{1}|P_{0})$. At the same time, it randomly chooses a leaving residue ($R_{0}$) from $P_{0}$ with the probability $\gamma (R_{0}|P_{0})$. The probability *p* to choose ($P_{0}$–$R_{0}$, $R_{1}$) is $\pi \left(P_{0}\right)\lambda \left(R_{1}|P_{0}\right)\gamma \left(R_{2}|P_{0}\right)$, and the acceptance can be calculated through Equation (10). Reprinted with permission from Rao et al. [101]. (**d**) Vitrimer model. Illustration of the two-step metathesis reactions in the vitrimer system. Illustration of the (**e**) heterogeneous dilute and (**f**) homogeneous dense systems of the vitrimers. Reprinted with permission from Lei et al. [119].

One way to simulate the formation and breakage of reversible bonds is by using the MC approach [52]. Crosslinking reactions are implemented by trial moves that reconstruct chain segments and attempt to create a dimerization reaction between pairs of reactive sites [118]. This model can be parameterized by the reaction equilibrium constant, obtained by experiments or atomistic/quantum simulations of the reactive species being free in a solution [118]. However, such an algorithm does not take into account the existence of multiple equilibria and the detailed balance [101,119,120] when simulating multivalent, multi-species systems [101]. The detailed balance of the bond swap scheme (Figure 15) is given by Equation (9) [101]:

(9) $$\frac{p/\nu^{u}acc}{p^{\prime }/\nu^{\prime u}acc^{\prime }}=\exp\left(-\beta \Delta G_{0}\right)$$

where acc and $acc^{\prime }$ are the acceptance of an MC move for the bond swap, and, for the forward and reverse proposals, $\beta =1/k_{B}T$, and $\Delta G_{0}$ is the reaction’s free energy change. Most existing bond swap algorithms assume the bond swap pair process is symmetric, implying $\frac{p^{\prime }/\nu^{\prime u}}{p/\nu^{u}}=1$, where *p* is the probability of a bond swap, and $\nu^{u}$ is the total unoccupied valence of the attacking residue [101].

The terms $p^{\prime }$ and $\nu^{\prime u}$ correspond to the reverse bond swap. However, this may not be true for multivalent and multi-species systems [101]. Thus, a bias term for the acceptance of each MC move, for the bond swap, is introduced, and, according to the Metropolis–Hastings rule [76,111], an MC move takes the form of Equation (10) [101].

(10) $$acc=\left(1,\frac{\nu_{1}^{u}}{\nu_{0}^{u}+1}\exp\left(-\beta \Delta G_{0}\right)\right)$$

The MC–proposed algorithm (Figure 15a–c) appears to have good quantitative agreement with theoretical predictions for an ideal monovalent diatomic system, an ideal multivalent linker system and a binary chain system [101]. The scheme can also be easily parallelized and implemented in hybrid MD–MC. In this case, the acceptance of an MC move, for the bond swap, is then Equation (11) [101], where $\Delta G_{a}$ is an activation energy that may be tuned to control the kinetics of bond swaps [101].

(11) $$acc=\exp\left(-\beta \Delta G_{a}\right)\left(1,\frac{p^{\prime }/\nu^{\prime u}}{p/\nu^{u}}\exp\left(-\beta \Delta G_{0}\right)\right)$$

While the efficiency of the MD–MC algorithm is reduced with the rigidity of the bond, it does not introduce an effective repulsion at high densities, like the three-body potential [101], which may affect the thermodynamics of the system. A hybrid MD–MC algorithm does not introduce any artificial potential in MD simulations [101].

Entropy has been found to play a dictating role in linker-mediated vitrimers [119], in agreement with the mean field theory [119,120]. It was assumed that the metathesis reaction (another type of associative exchange reaction) consisted of two steps, as shown in Figure 15d–f. In particular, the heterogeneity of reactive sites could result in a mismatch of reaction rate constants for the same metathesis reaction in the first and second crosslinking steps [119]. In addition, the increase in crosslinker concentration could induce a reentrant gel-sol transition in the vitrimer, which could alter the mechanical, reshaping and recycling behavior of the system, even in the absence of a temperature change [119,121]. At low temperatures, the extent of the crosslinking of the system could be effectively tuned by the concentration of crosslinkers [119], which could be an issue in experiments [121,122], since lowering the temperature would not lead to highly crosslinked networks [119]. A thermo-gelling vitrimer, which remained liquid because of the addition of inhibitors that prevented crosslinking at low temperatures, and which underwent entropy-driven crosslinking occurring with an increasing temperature, was simulated by MC [120]. These coarse-grained simulations agreed with the mean field theory that describes crosslinking above the gas–liquid phase separation temperature as being induced by short-range van der Waals attraction [120]. At high temperatures, the vitrimers were always crosslinked because of entropy, and mechanical properties could be tuned reversibly in situ by changing the activation barrier using catalysts adjusting the concentration of inhibitor molecules [120] or altering the crosslink density [65].

## 4. Hybrid Molecular Dynamics/Monte Carlo

In this section, we discuss studies that focus on CAN and vitrimer behavior implemented by a hybrid MD–MC approach [111,123,124,125,126,127,128,129] using only coarse-grained models (Kremer–Grest (KG) bead-spring model [130]), since we are unaware of any atomistic simulation efforts along these lines at the time of writing. This hybrid approach can be traced back to the introduction of the end-bridging MC move mechanism by Pant and Theodorou [131]. Similar algorithms that combine MD and MC methods were applied following that work [131] in supramolecular polymers [127], thermoreversible gels [132] and telechelic polymers [76,133,134].

It is shown that BERs change the diffusion mode of the vitrimers’ constituent molecules, which affects BER and relaxation dynamics [111]. The relaxation dynamics also features two regimes. Fast relaxation is not affected by the BER activation energy barrier, and slow relaxation has a relaxation time dependent on the BER activation energy [111]. This indicates that high values of the BER activation energy are a limiting factor of phase transition, since the lifetime [29] of the dynamic bond is longer than the structural relaxation time [111]. The dynamical relaxation dynamics in vitrimers with a fast bond exchange rate followed WLF and Arrhenius-like equation behaviors at low and high temperatures, respectively [125], which is consistent with experimental observations for epoxy vitrimers [36] and poly(hexyl methacrylate) vitrimers [135]. Through an implementation of an artificial parameter, it is possible to scale the energy difference between two states in the MC step, and the activation energy of the BER can be controlled [124].

**Figure 16:**
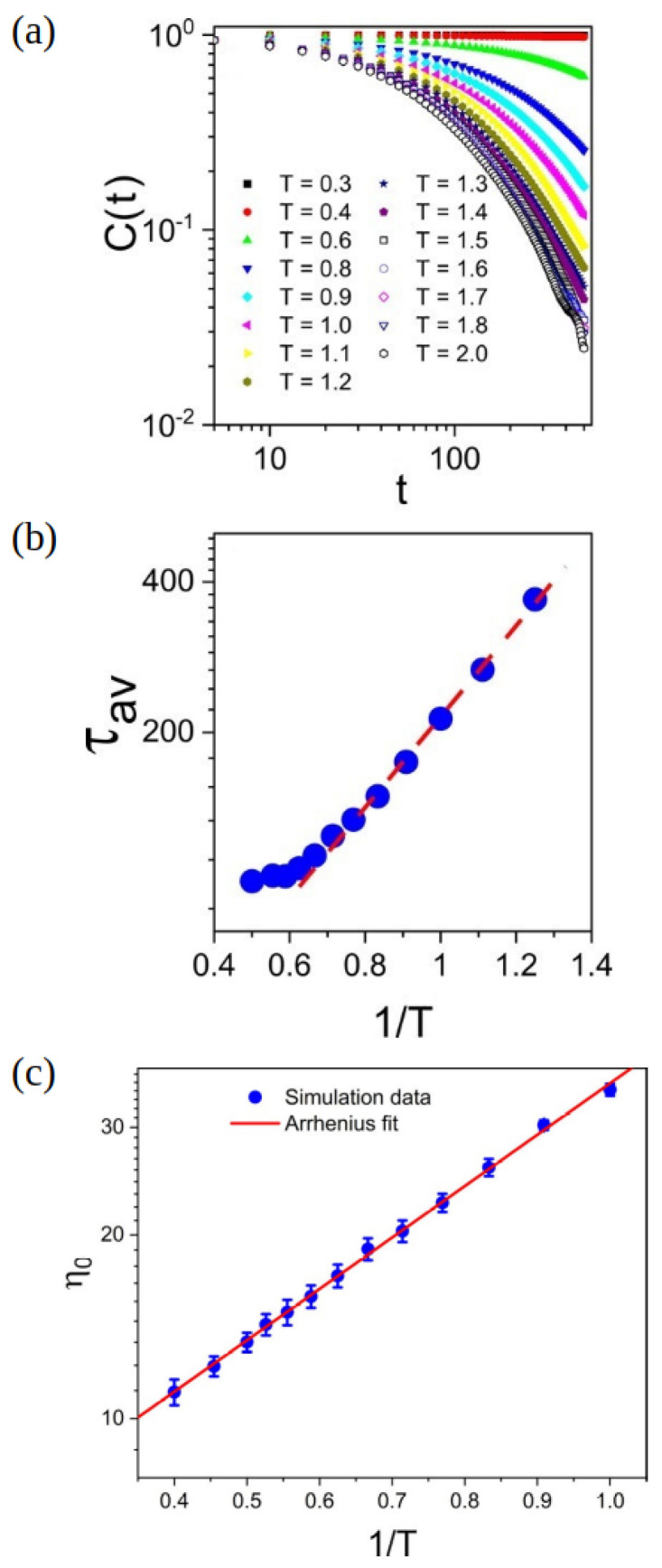
(**a**) Autocorrelation function of dynamic bonds. C(t) is a function of simulation time at different temperatures. *t* is in reduced units, having its units reduced by $\sigma_{LJ}\sqrt{m/\epsilon_{LJ}}$. (**b**) Average lifetime $\tau_{av}$ of bonds as a function of temperature. The dashed red line shows the Arrhenius fit to the data. The values of $\tau_{av}$ are determined from the decay timescale of C(t) in (**a**) at different temperatures. $\tau_{av}$ and *T* are in reduced units, having their units reduced by $\sigma_{LJ}\sqrt{m/\epsilon_{LJ}}$ and $\epsilon_{LJ}/k_{B}$, respectively. (**c**) Zero-shear viscosity of vitrimer networks at elevated temperatures, $T>T_{\nu }$, as a function of reciprocal temperature. The red line shows the Arrhenius fit to the data. The values of the zero-shear viscosity are obtained from the shear viscosity curves at each temperature using NEMD simulations. The shear viscosity $\eta_{0}$ in that case is dimensionless. Reprinted with permission from Perego and Khabaz [123].

The coarse-grained model vitrimer showed two transition temperatures [123] and a specific volume similar to thermosets at a low temperature [123]. In addition to the conventional $T_{g}$, $T_{\nu }$ (determined by the thermal expansion coefficient [123]), which is observed in vitrimers, is also detected by the coarse-grained model and corresponds to changes in the network topology at higher temperatures [123]. The model vitrimer shows a terminal regime at low frequencies, which characterizes the fluidity and stress relaxation of these networks [123]. The concentration of active beads does not seem to have an impact on the relaxation mode. Increasing the temperature can change network topology rearrangement from a diffusion-controlled regime [136,137] to a reaction-controlled regime [111]. The values of the bond lifetime autocorrelation function C(t) as a function of time at different temperatures are shown in Figure 16a [123]. In particular, at low temperatures ($T\;\leq \;T_{g}$), the BER is slow, therefore the C(t) function does not decay and shows a value close to unity (permanent crosslinked bonds). As the temperature increases, C(t) shows a stretched exponential decay and drops faster at higher temperatures. At very high temperature, the C(t) behavior becomes independent of temperature. The behavior of C(t) is best described by fitting the autocorrelation data to a stretched exponential function $C\left(t\right)=exp(-{\left(t/\tau \right)}^{\theta })$, where θ is the stretching exponent and τ is the relaxation time. An average value of θ of 0.84 is shown at all temperatures, which reveals that the exchange relaxation process is close to an exponential decay [123]. Moreover, the average lifetime is calculated based on the relation $\tau_{av}=\int_{0}^{\infty }C\left(t\right)dt=\frac{\tau }{\theta }\Gamma \left(\frac{1}{\theta }\right)$, where Γ is the Gamma function [123]. The average lifetime of the dynamic bonds at moderate to high temperatures (T≤1.7, in reduced units of $\epsilon_{LJ}/k_{B}$) followed an Arrhenius-like temperature dependence (Figure 16b) in agreement with other works [95,110,114,116], including reports using a coarse-grained vitrimeric model implemented with the three-body potential [99,110]. At very high temperatures (T≥1.7, in reduced units of $\epsilon_{LJ}/k_{B}$), the lifetime of the bonds becomes temperature-insensitive and shows a limiting value of around 100.

To confirm that the viscosity of the simulated vitrimer follows Arrhenius-like behavior, at temperatures above the topology freezing temperature ($T\;\geq \;T_{\nu }$), the zero-shear viscosity $\eta_{0}$ was determined by extrapolation of the shear viscosity to a zero-shear rate [123]. An Arrhenius plot of $\eta_{0}$ is depicted in Figure 16c and described by the relation $\eta_{0}=5.09exp\left(\frac{1.94}{T}\right)$, meaning that, as the temperature increases, the viscosity decreases exponentially [123,129]. This is closely related to BERs whose frequency increases with temperature. It is also noteworthy that this behavior of the zero-shear viscosity is consistent with the temperature dependence of the shift factors at $T\;\geq \;T_{\nu }$. By using the TTSP principle, it is possible to extend the frequency range of rheology, by simulations, from 3 to 10 orders of magnitude, in the terminal regime, as seen in experiments [138,139], capturing the elastic modulus [123]. Expanding on this point, the poly(dimethylsiloxane) (PDMS) vitrimer (which has a flexible backbone) showed an Arrhenius temperature dependence for $\eta_{0}$, while a poly(styrene) vitrimer (which has a rigid backbone) only shows Arrhenius behavior at high temperature [140]. Simulations reveal that the lifetime of the dynamic bonds determine the rheology and dynamics of these networks. When the rate of the deformation is higher than the rate of the bond exchange, the system behaves as a typical thermoset, while at lower rates, the vitrimer behaves as a viscous liquid [123]. In particular, it is shown that the dynamic and mechanical properties of the vitrimers are strongly affected by the number of successful bond exchanges [124]. A higher number of exchanges (achieved, for instance, by lowering the energy barrier of the bond exchange) results in greater deformation before fracture [124].

Moreover, it was shown that changing the crosslink fraction in a hybrid MD–MC coarse-grained vitrimeric system where a vacuum interface is present impacts the bulk polymer’s density [141]. The dynamic bonding was handled by the MC Metropolis algorithm, where the equilibrium fraction of crosslinks was determined by their chemical potential μ. The crosslink fraction was independent of polymer chain length at a fixed μ, but increased with μ. Bulk polymer density also increased with the crosslink fraction [141]. It should also be noted that, although the crosslinks are well-distributed in the bulk, at equilibrium, they were depleted at the interface [141]. These findings were interesting because of the lack of clear and concise literature on the impact of crosslinks on polymer density and, more specifically, that of dynamic crosslinks on saturated polymer density [141]. Since no significant variation in the system energetics was observed, it was implied that the increased cohesion (due to an increase in crosslink fraction) was entropic in nature [141]. Thus, density seemed to be a key property in tuning vitrimer behavior, such as their miscibility [141,142].

A more homogeneous stress distribution was observed in vitrimers before failure than in a comparable thermoset network [124]. Intermediate regimes of stress relaxation modulus ($G\left(t\right)\;\approx \;t^{1/2}$) and shear creep compliance ($J\left(t\right)\;\approx \;t^{1/2}$, where $J\left(t\right)=\frac{\epsilon }{\sigma }$) in creep were also detected [125,143]. Vitrimers reached an equilibrium creep compliance, where creep was no longer suppressed, and the material continued to deform under a shear load, indicating that stress relaxation events were taking place within the network [143]. The transient network model of Fricker actually associates terminal relaxation with the relation $G\left(t\right)\;\approx \;t^{1/2}$ [144]. Moreover, shear compliance and the influence of deformation on dynamics were further investigated [126]. It was shown that the crosslinker mobility was enhanced for both glassy vitrimers and a typical thermoset network. However, while the thermoset showed a plateau in shear compliance over long times, the vitrimer was able to achieve much higher shear compliance over long times [126]. This was due to the fact that vitrimers can readily accommodate deformation and change their topology. Enhanced homogeneous motion was also observed in the vitrimer. The topology and dynamics of vitrimers were explained in terms of deformation, and, further, a stage of deformation was detected, compared with the thermoset [126].

Moreover, using a coarse-grained (KG) model of dangling chains (linear polymers with N=10 or N=2) attached to a polymer network by effective chains fixed in space at one end, and containing associating groups (stickers) on the other end, Stukalin et al. investigated self-healing behavior [76]. The stickers acted as a convenient theoretical concept representing the instantaneous orientation of the binding sites on a reversible bond or a polymer segment. They could be modeled as beads attached to their host; that is, a reversible crosslink or polymer segment [52]. The stickers could form reversible bonds as pairs with other stickers [145]. The thermal activation of sticky bond dissociation suggested exponential behavior over the lifetime of the sticky bond ($\tau_{SB}$), $\frac{1}{\tau_{SB}}\propto exp\left(-\Delta E_{sw}/k_{B}T\right)$ [127,146,147], in alignment with Kramer’s rate theory [148]. Sticky bonds were formed and broken using the Metropolis MC criterion. In order to investigate the self-healing mechanism, an “ideal–cut” was introduced along the xy plane through the middle of the simulation cell. After this “ideal–cut”, MC updates of sticky bonds were switched on, but during the waiting period, no reversible bonds were allowed to form across this fracture plane [76]. After some waiting time, fractured sections were shifted toward each other by half lengths of the simulation cell along the fracture plane in the x direction. MC updates for the whole system were switched off during this shift. After the shift, the confining potential was turned off, the MC updates of dynamic bonds were switched on, and the chains were allowed to interpenetrate and form sticky bonds (bridges) across the fractured interface, providing information concerning denoting the healing period [76]. Although the timescale for this model network to equilibrate was still “very short” compared with those observed in experiments of self-healing supramolecular rubbers [149], this model captured the molecular picture of self-healing reversible networks and shed some light on the mechanism of the self-healing process [76].

Furthermore, the structural, dynamical and linear rheological behavior of unentangled side-chain-linked vitrimers were also studied at a single crosslink density, recently, with such an MD–MC hybrid approach and coarse-grained model, by Xia et al. [129]. That simulated system [129] resembled an experimental PDMS vitrimer [150]. A minor variation was found in the topology as a function of the temperature [129], showing that this microscopic picture was consistent with experiments by Porath et al. [151]. Through the analysis of the relaxation time of the dynamic bonds and the incoherent intermediate scattering function ($F_{s}\left(q,t\right)$) of the reactive beads [129], the topological freezing transition temperature of $T_{\nu }=0.6$ (in reduced units of $\epsilon_{LJ}/k_{B}$) was determined. As the temperature approached $T_{\nu }$, the bond exchange behavior and the system dynamics showed dynamic heterogeneity. The dynamics followed a combination of Arrhenius and WLF-like behaviors at temperatures lower and higher than $T_{\nu }$, respectively [129]. However, that physical picture revealed by such MD–MC simulations [129] was different from the one from the experimental results on ethylene vitrimers by Soman et al. [152]. In ethylene vitrimer experiments [152], the non-Arrhenius behavior emerged at lower experimental temperatures ($T_{g}/T>0.85$) [95,152] and the exchange time crossover was attributed to the glassy segmental relaxation on the bond exchange rather the topological freezing temperature [129]. It was worth noting that such hybrid MD–MC simulations [129,146] could be performed in weakly supercooled states with low energy barriers, whereas deeply supercooled states with high activation energy were probed in experiments [95].

**Figure 17:**
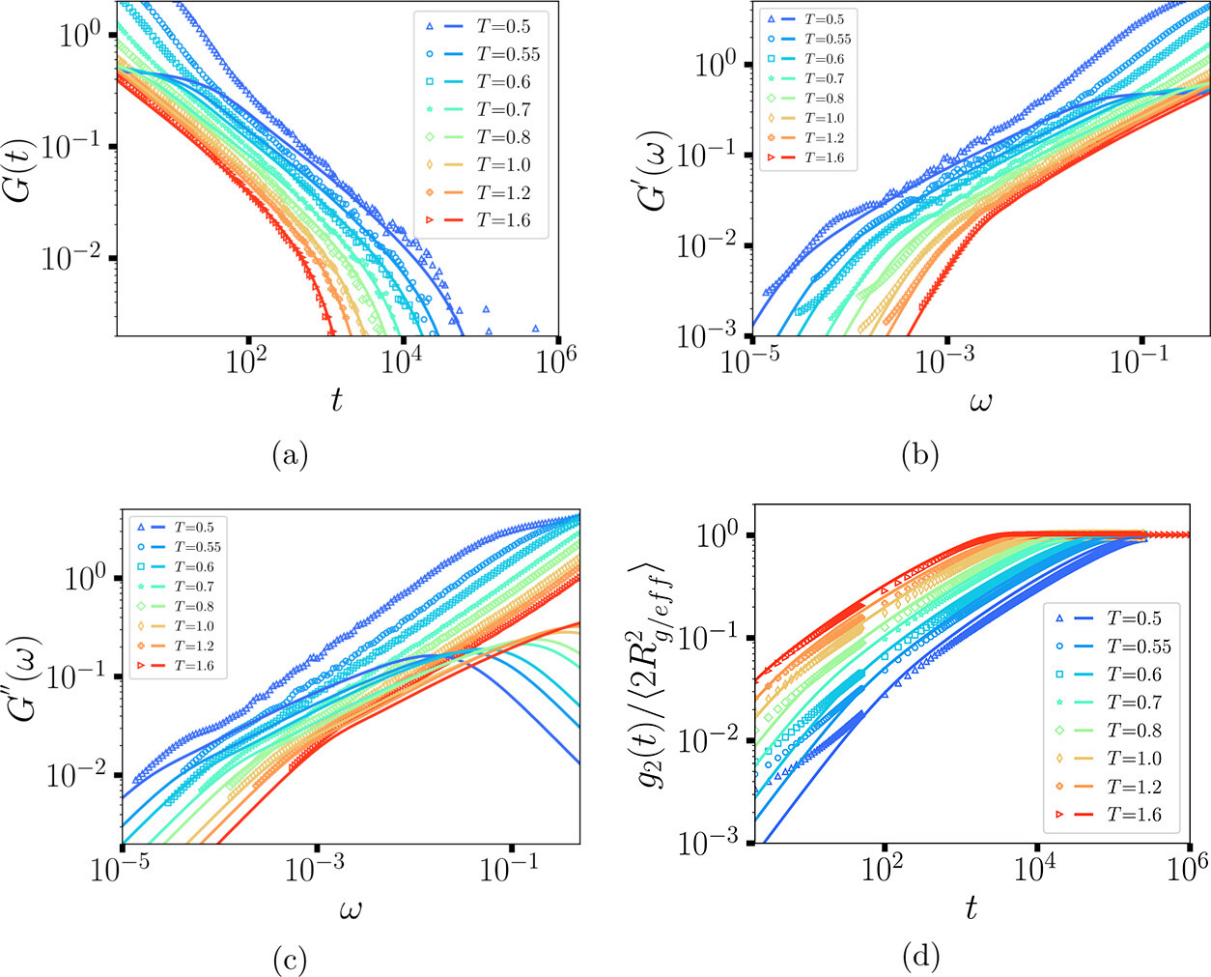
Comparison between the simulated results and the theoretical values of the SRM for (**a**) the stress relaxation modulus G(t), (**b**) the elastic modulus $G^{\prime }\left(\omega \right)$, (**c**) the loss modulus G″(ω) and (**d**) the reduced mean square displacement of segments relative to the effective mass center of a chain, $g_{2}\left(t\right)/<2R_{g/eff}^{2}>$, at different temperatures, where the symbols are the simulation results and the solid lines are the predicted values of the SRM by analyzing each chain without introducing any fitting parameters. *t* has its units reduced by $\sigma_{LJ}\sqrt{m/\epsilon_{LJ}}$ and ω, G(ω), $G^{\prime }\left(\omega \right)$, G″(ω) as well as $g_{2}\left(t\right)/<2R_{g/eff}^{2}>$ are dimensionless. Reprinted with permission from Xia et al. [129].

The relaxation moduli and the diffusion of segments relative to the chain center of mass were predicted based on the values of the relative effective friction coefficient of the sticky beads. Quantitative agreement was observed between the simulation and theoretical results, from the sticky Rouse model (SRM) [153] for the relaxation of the shear and elastic moduli (G(t) and $G^{\prime }\left(\omega \right)$, respectively) and reduced mean square displacement of segments relative to the chain center of mass $g_{2}\left(t\right)/<2R_{g/eff}^{2}>$ (Figure 17), where $g_{2}\left(t\right)$ is the mean square displacement and $R_{g/eff}$ is the radius of gyration, especially at the intermediate and long times/low frequencies [129].

In particular, for the vitrimer at high temperatures (T=1.6, in reduced units of $\epsilon_{LJ}/k_{B}$), the simulation results were in agreement with the SRM predictions, as can be seen in Figure 17a,b,d. By decreasing the temperature, the glassy behavior becomes more significant [129]. A scaling master curve is obtained for the zero-shear viscosity and the Rouse relaxation time [129]. The Rouse regime, where there is a scaling of $t^{-0.5}$ before the terminal relaxation, shifts to lower frequencies (longer times). A fair agreement is found between the simulation predictions and the theory at a long time/low frequency.

It is concluded that, given the quantitative agreement between simulations and theory, not only in the work by Xia et al. [129] but also in the work of physical associative polymers by Jiang et al. [154], that the associative mechanism has little effect on the effectiveness of the SRM in describing the linear viscoelastic behavior of associative polymer networks [129,132]. The terminal relaxation of the loss modulus $G^{\prime \prime }\left(\omega \right)$ is also predicted by the SRM, which is originally proposed for the transient polymer network connected by physical interactions and was validated for physical associative polymers [154]. This approach is also used by Ricarte and Shanbhag to elucidate structure–viscoelasticity property relations for unentangled vitrimer melts [140]. In particular, two different versions of the SRM are explored: the simplified sticky Rouse (SSR) and the inhomogeneous Rouse (IHR) model, which is a generalization of the Rouse model [140]. The IHR model (but not the SSR) accounted for interactions between slow modes that arise due to crosslinking and fast Rouse modes of the underlying polymer chain. In a bead-spring unentangled chain (with *N* beads) of which $N_{x}$ beads were sticky, as $N_{x}$ increased, the rheological response of the SRM was expected to yield a result statistically indistinguishable from uniform sticky beads; in block configurations, however, the behavior is different, and the relaxation happens faster than in random or uniform distributions [140].

The two different types of CANs, being associative and dissociative, are simulated differently because of their bond exchange mechanism [147,155]. Dissociative CANs are represented simply by two separate MC events, which are the breaking and formation of the bond. Each of those events have a specific, unrelated probability associated with them. The dissociative CANs also only need two stickers or crosslinkers inside a “cut-off” radius that are either bonded or open [146] in order to break or create a bond, respectively [155]. For associative CANs, the BER happens in a single MC step, by bond exchange, since the crosslinking density remains constant. They are also implemented under the assumption that no stickers or crosslinkers are open at all times. Associative CANs also need two pairs of bonded stickers [146], or crosslinkers, within the cut-off radius for the bond exchange to happen [155]. Moreover, dissociative and associative CANs do not interact with the same energy parameters. As a general case, if two sticky monomers are bonded, they interact via a “sticky bond” potential [76,127,155], $U_{SB}$, which is a modified form of the standard covalent FENE potential, as shown in Equation (12) [127,155]:

(12) $$U_{SB}\left(r,\epsilon_{LJ}\right)=U_{FENE}-U_{FENE}\left(b_{0}\right)-H$$

The distance-independent energy offset $U_{FENE}\left(b_{0}\right)-H$ ($b_{0}$ is the equilibrium FENE bond length) is used to control the fraction of closed bonds in simulations. The parameter *H* represents the sticky binding energy for dissociative CANs [156]. However, for associative CANs, *H* is an additional parameter that modulates the reaction’s degree of difficulty [155]. Associative CANs exhibit strong mechanical strength and creep resistance, making them suitable for self-adhesion materials [147]. On the other hand, the presence of open stickers in dissociative CANs drives the healing through walking diffusion across the damaged surface [147]. Dissociative CANs have a shorter self-healing timescale compared with the self-adhesion timescale of associative CANs [147].

**Figure 18:**
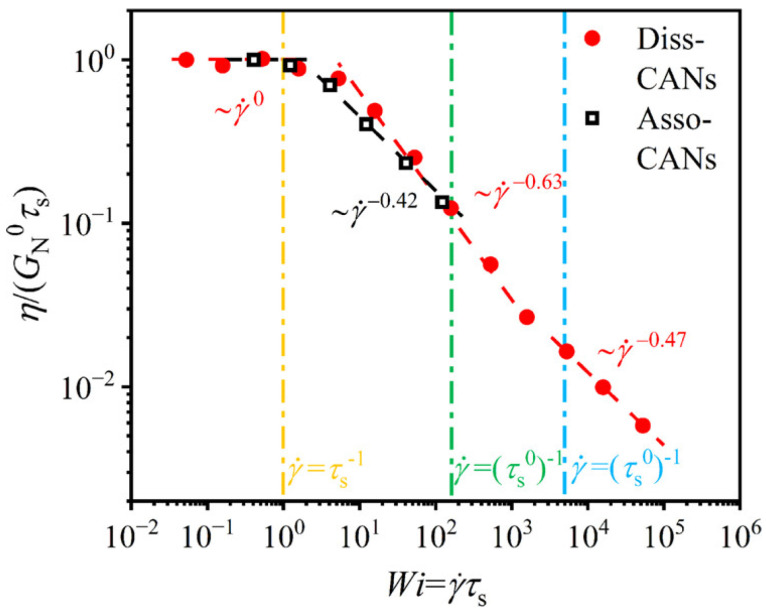
Shear thinning of dissociative CANs (red circle) and associative CANs (black empty squares). The steady viscosity is normalized by $G_{N}^{0}$ and $\tau_{s}$. Dot-dashed lines represent the characteristic shear rates for dissociative CANs (yellow $\tau_{0}^{-1}$ and blue ${\left(\tau_{s}^{0}\right)}^{-1}$) and associative CANs (yellow $\tau_{0}^{-1}$ and green ${\left(\tau_{s}^{0}\right)}^{-1}$). Both $\eta /(G_{N}^{0}\tau_{s})$ and $W_{i}=\dot{\gamma }\tau_{s}$ are dimensionless. Reprinted with permission from Cui et al. [155].

In addition, the different CANs have similar linear viscoelastic behavior but show dissimilarities with respect to nonlinear viscoelasticity [155]. Nonetheless, during linear viscoelasticity, the crosslink density increases for dissociative CANs with a decreasing temperature or increasing kinetic activation energy [155]. This denotes an enhancement of sticker movements and a decrease in the prefactor $\tau_{s}^{0}$ of the characteristic terminal relaxation time, $\tau_{s}$, when expressed in the Arrhenius form, which is related to the Rouse relaxation [155]. In comparison, for associative CANs, $\tau_{s}^{0}$ is constant with respect to the kinetic activation energy since the crosslink density is constant [155]. We see in Figure 18 the steady viscosity $\eta /(G_{N}^{0}\tau_{s})$ (during steady shear) with respect to $W_{i}=\dot{\gamma }\tau_{s}$ (where $G_{N}^{0}$ is the plateau modulus and $\dot{\gamma }$ is the shear rate) [155]. We can notice in Figure 18 that dissociative CANs display a wider range of $W_{i}$ than associative CANs, as well as three scaling relationships instead of two [155]. In the range of $\dot{\gamma }<\tau_{s}^{-1}$, both CANs show that η is constant with $W_{i}$. For $\tau_{s}^{-1}<\dot{\gamma }<{\left(\tau_{s}^{0}\right)}^{-1}$, stickers begin dissociating for dissociative CANs, and BER accelerates for associative CANs [155]. Lastly, in the region of $\dot{\gamma }>{\left(\tau_{s}^{0}\right)}^{-1}$, the chains do not have enough relaxation time for the magnitude of the shear rate [155]. For dissociative CANs, this results in a slower shear thinning, and for associative CANs, since the stickers cannot dissociate, the network cannot support much shearing before becoming rigid, resulting in a weaker shear thinning [155]. The lifetime of dynamic crosslinks in associative CANs is also consistently longer than those in dissociative CANs [155], and they show greater strain hardening [72,155]. The discrepancies for shear thinning and strain hardening can be explained with the fact that associative CANs can only have bridge- and loop-type chains, while dissociative CANs can also produce free and dangling chains [155]. Dissociative CANs are thus able to show orientation-induced dissociation, which strengthens shear thinning [155]. These chains can then orient themselves and reform bridge chains periodically while undergoing a tumbling process, which weakens strain hardening [155]. Limits in the nonlinear application of associative CANs show that ${\left(\tau_{s}^{0}\right)}^{-1}$ is the upper limit for the shear rate, since the BER cannot happen beyond that point [155].

## 5. Conclusions

In this review, we focused on molecular simulation (by MD, MC and a hybrid MD–MC method) studies of CANs and vitrimers. In particular, the structure, dynamics, and mechanical and rheological behavior of unentangled vitrimers were investigated using both atomistic and coarse-grained polymer models.

Through atomistic simulations, it is shown that the stress relaxation occurs as the BERs continue. At low temperatures, the BERs slow down and the vitrimer behaves as an elastomer. Although stress can deform the vitrimer, BERs can return the network to its isotropic state. It was shown that creating DA networks with more flexible chains would lead to a lower $T_{g}$, lower strength and faster shape recovery. The degree of network anisotropy is proportional to the amount of creep strain. Different creep regimes can be simulated by atomistic modeling, revealing the void growth during deformation. The atomistic models generated by both classical and ReaxFF are able to confirm the self-healing ability of vitrimers. The self-healing ability is more effective at a higher crosslink density and at temperatures above $T_{\nu }$ where BERs are more frequent. ML FFs are not extensively developed for CANs or vitrimers, except for polyimine–imine networks, which are able to reproduce reaction kinetics and transition state geometries that cannot be predicted by classical FF. The inverse design of bifunctional transesterification vitrimers can be applied by BO using $T_{g}$ as a target.

On the other hand, although coarse-grained models do not contain any chemical detail information, they can be used to reveal the polymer physics of dynamic CANs and vitrimers by consistently modeling the bond-swapping mechanism. Although MD simulations incorporating a three-body potential have an advantage over MC moves since, in the former case, the dependence of exchange probability on physical parameters such as the bond force arises naturally, MC is advantageous for simulating BERs, since the three-body potential may misbehave at high densities, where interactions between more than two particles are frequent. The two transition temperatures ($T_{g},T_{\nu }$) of vitrimers are observed via coarse-grained models. Bulk polymer density is key to tuning vitrimer behavior. Moreover, segmental dynamics are faster than polymer chain dynamics and bond swap dynamics, which have an increased energy barrier. The chain segment relaxation follows the VFT equation and an Arrhenius equation at low and high temperatures, respectively. In addition, it was shown that the lifetime of the BERs, self-healing times and zero-shear viscosities of vitrimer networks follow an Arrhenius dependence with the temperature. Both dynamic and mechanical properties are strongly affected by the number of successful bond exchanges, with storage and loss moduli decreasing at high temperatures due to low numbers of dynamic covalent bonds and lifetimes. The simulated shear and elastic moduli agree with the theoretical results of the SRM at the intermediate and long times/low frequencies. The lifetime of dynamic crosslinks in associative CANs is also consistently longer than those in dissociative CANs, showing a greater strain hardening, mechanical strength and creep resistance. Associative CANs are suitable for self-adhesion materials whereas dissociative CANs are suitable for self-healing materials. The different CANs have a similar linear viscoelastic behavior, but show dissimilarities regarding nonlinear viscoelasticity. The discrepancies in strain hardening and shear thinning between the two types of CANs are explained by the fact that associative CANs can only have bridge- and loop-type chains, while dissociative CANs can also produce free and dangling chains.

However, in experimental CANs or vitrimers, there are other factors that have not yet been explored and taken into consideration in molecular simulations that can influence dynamic crosslinking. These include, for example, different crosslink densities, the semi-crystallinity of the crosslinked polymer chains, their polydispersity, the van der Waals attraction between different chemical species, additives and the inclusion of nanofillers (the exception being those studies that included GO and nanorods). In addition, no atomistic simulation study included catalysts, though catalytic species are frequently used to modulate BER kinetics, tuning the mechanical properties by altering the activation barrier. Moreover, the regime of entangled associating polymer networks is completely untouched by computer simulations. Nevertheless, the molecular simulation methodologies discussed in this work are appropriate tools not only to investigate these factors in the structure and dynamics, mechanical and rheological response of dynamic networks, but also to reveal the physical mechanisms that govern their behavior. 

## Data Availability

Data are contained within the article.
